# Exploration of multi‐target effects of 3‐benzoyl‐5‐hydroxychromen‐2‐one in Alzheimer’s disease cell and mouse models

**DOI:** 10.1111/acel.13169

**Published:** 2020-06-04

**Authors:** Te‐Hsien Lin, Ya‐Jen Chiu, Chih‐Hsin Lin, Chung‐Yin Lin, Chih‐Ying Chao, Yu‐Chieh Chen, Shu‐Mei Yang, Wenwei Lin, Hsiu Mei Hsieh‐Li, Yih‐Ru Wu, Kuo‐Hsuan Chang, Guey‐Jen Lee‐Chen, Chiung‐Mei Chen

**Affiliations:** ^1^ Department of Life Science National Taiwan Normal University Taipei Taiwan; ^2^ Department of Neurology, Chang Gung Memorial Hospital Chang Gung University College of Medicine Taoyuan Taiwan; ^3^ Medical Imaging Research Center, Institute for Radiological Research Chang Gung University/Chang Gung Memorial Hospital Taoyuan Taiwan; ^4^ Department of Chemistry National Taiwan Normal University Taipei Taiwan

**Keywords:** Alzheimer's disease, apoptosis, chaperone, LM‐031, oxidative stress, Tau

## Abstract

Microtubule‐associated protein Tau, abundant in the central nervous system (CNS), plays crucial roles in microtubule assembly and stabilization. Abnormal Tau phosphorylation and aggregation are a common pathogenic hallmark in Alzheimer's disease (AD). Hyperphosphorylation of Tau could change its conformation and result in self‐aggregation, increased oxidative stress, and neuronal death. In this study, we examined the potential of licochalcone A (a natural chalcone) and five synthetic derivatives (LM compounds) for inhibiting Tau misfolding, scavenging reactive oxygen species (ROS) and providing neuroprotection in human cells expressing proaggregant ΔK280 Tau_RD_‐DsRed. All test compounds were soluble up to 100 μM in cell culture media and predicted to be orally bioavailable and CNS‐active. Among them, licochalcone A and LM‐031 markedly reduced Tau misfolding and associated ROS, promoted neurite outgrowth, and inhibited caspase 3 activity in ΔK280 Tau_RD_‐DsRed 293 and SH‐SY5Y cells. Mechanistic studies showed that LM‐031 upregulates HSPB1 chaperone, NRF2/NQO1/GCLC pathway, and CREB‐dependent BDNF/AKT/ERK/BCL2 pathway in ΔK280 Tau_RD_‐DsRed SH‐SY5Y cells. Decreased neurite outgrowth upon induction of ΔK280 Tau_RD_‐DsRed was rescued by LM‐031, which was counteracted by knockdown of NRF2 or CREB. LM‐031 further rescued the downregulated NRF2 and pCREB, reduced Aβ and Tau levels in hippocampus and cortex, and ameliorated cognitive deficits in streptozocin‐induced hyperglycemic 3 × Tg‐AD mice. Our findings strongly indicate the potential of LM‐031 for modifying AD progression by targeting HSPB1 to reduce Tau misfolding and activating NRF2 and CREB pathways to suppress apoptosis and promote neuron survival, thereby offering a new drug development avenue for AD treatment.

## Graphical abstract

Through upregulating HSPB1 chaperone to reduce Tau misfolding, and enhancing NRF2 and CREB pathways to reduce ROS and apoptosis in ΔK280 TauRD‐DsRed SH‐SY5Y cells, as well as promoting neuron survival and cognitive function in hyperglycemic 3×Tg‐AD mice, LM‐031 (3‐benzoyl‐5‐hydroxychromen‐2‐one) displays potential for Alzheimer’s disease treatment.

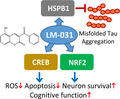

## 1 INTRODUCTION

Several neurodegenerative disorders known as tauopathies, including Alzheimer's disease (AD), are characterized by abnormal aggregation of Tau protein (Iqbal et al., 2005). In these diseases, Tau becomes abnormally hyperphosphorylated and misfolded, forming insoluble aggregates. Phosphorylation of Tau has been proposed as the link between oxidative stress, mitochondrial dysfunction, and connectivity failure (Mondragón‐Rodríguez et al., 2013). Chaperones and the ubiquitin–proteasome system, playing a defense role to remove misfolded proteins and rescue the neurons, are involved particularly in the early stage of tauopathies (Sherman & Goldberg, 2001). Thus, identification of potential Tau misfolding inhibitors could be a strategy for disease progression modification in these neurodegenerative diseases.

Encoded on chromosome 17, Tau is mainly expressed in neuronal axons, where they play an important role in microtubule dynamics and assembly, as well as in axonal transport and apoptosis (Lee, Goedert, & Trojanowski, 2001). Tau is a prototypical natively unfolded protein (Schweers, Schonbrunnhanebeck, Marx, & Mandelkow, 1994). Tau binds microtubules through C‐terminal repeat domain (Tau_RD_), which contains four highly conserved 18‐amino acid repeat domains (Goedert, Spillantini, Potier, Ulrich, & Crowther, 1989). The Tau_RD_ has been shown to reside at the core of paired helical filaments of neurofibrillary tangles (Goedert et al., 1989), one of the neuropathological hallmarks of AD, and a deletion mutant ∆K280 within Tau_RD_ (∆K280 Tau_RD_) has been found in patients with AD and other tauopathies (D’Souza et al., 1999; Momeni et al., 2009; Rizzu et al., 1999). Overexpressed ∆K280 Tau_RD_ in mouse neuroblastoma N2a cells is highly prone to being misfolded and forming aggregation (Khlistunova et al., 2006). Chemicals that prevent misfolding of Tau in the ∆K280 Tau_RD_‐expressing N2a cells are thought to be candidates for the treatment of AD and tauopathies (Pickhardt et al., 2005, 2007). Heat shock protein system that helps refolding of abnormal protein is also emerging as a key pathway for modulation to prevent AD‐related neurodegenerative damage (Trovato, Siracusa, Di Paola, Scuto, Fronte, et al., 2016; Trovato, Siracusa, Di Paola, Scuto, Ontario, et al., 2016). We have also established human cells expressing pro‐aggregated ∆K280 Tau_RD_ to screen herbal extracts or indole compounds exerting neuroprotection for AD and tauopathies treatment (Chang et al., 2016, 2017; Chen et al., 2018).

Licochalcone A is a major chalcone constituent obtained from the root of *Glycyrrhiza inflata* (Wang, Lee, & Wang, 2004). Licochalcone A has been reported to enhance NRF2 (nuclear factor, erythroid 2‐like 2)‐mediated defense mechanism against oxidative stress in spinocerebellar ataxia type 3 cell models (Chen, Weng, et al., 2014), mouse RAW 264.7 macrophages (Lv, Ren, Wang, Chen, & Ci, 2015), and primary human fibroblasts (Kühnl et al., 2015). Furthermore, previously we have shown that licochalcone A and derivative compound LM‐031 (3‐benzoyl‐5‐hydroxychromen‐2‐one) upregulated HSPB1 (heat shock protein B1), activated NRF2 and CREB (cAMP‐responsive element‐binding protein 1) pathways to reduce Aβ misfolding and reactive oxygen species (ROS), promoted neurite outgrowth, and inhibited acetylcholinesterase in Aβ cell models (Lee et al., 2018). Here, we examined the ability of licochalcone A and five derivative compounds to prevent ∆K280 Tau_RD_ aggregation and oxidation as well as to promote neuroprotection by using human cells expressing DsRed‐tagged ∆K280 Tau_RD_ (Chang et al., 2016). We also explored the potential of LM‐031 for AD treatment in streptozocin (STZ)‐induced hyperglycemic 3 × Tg‐AD mice. Our findings indicate that the synthetic LM‐031 is a potential candidate for the development of AD and tauopathy therapeutics.

## 2 RESULTS

### 2.1 Test compounds and IC_50_ cytotoxicity

**Figure 1:**
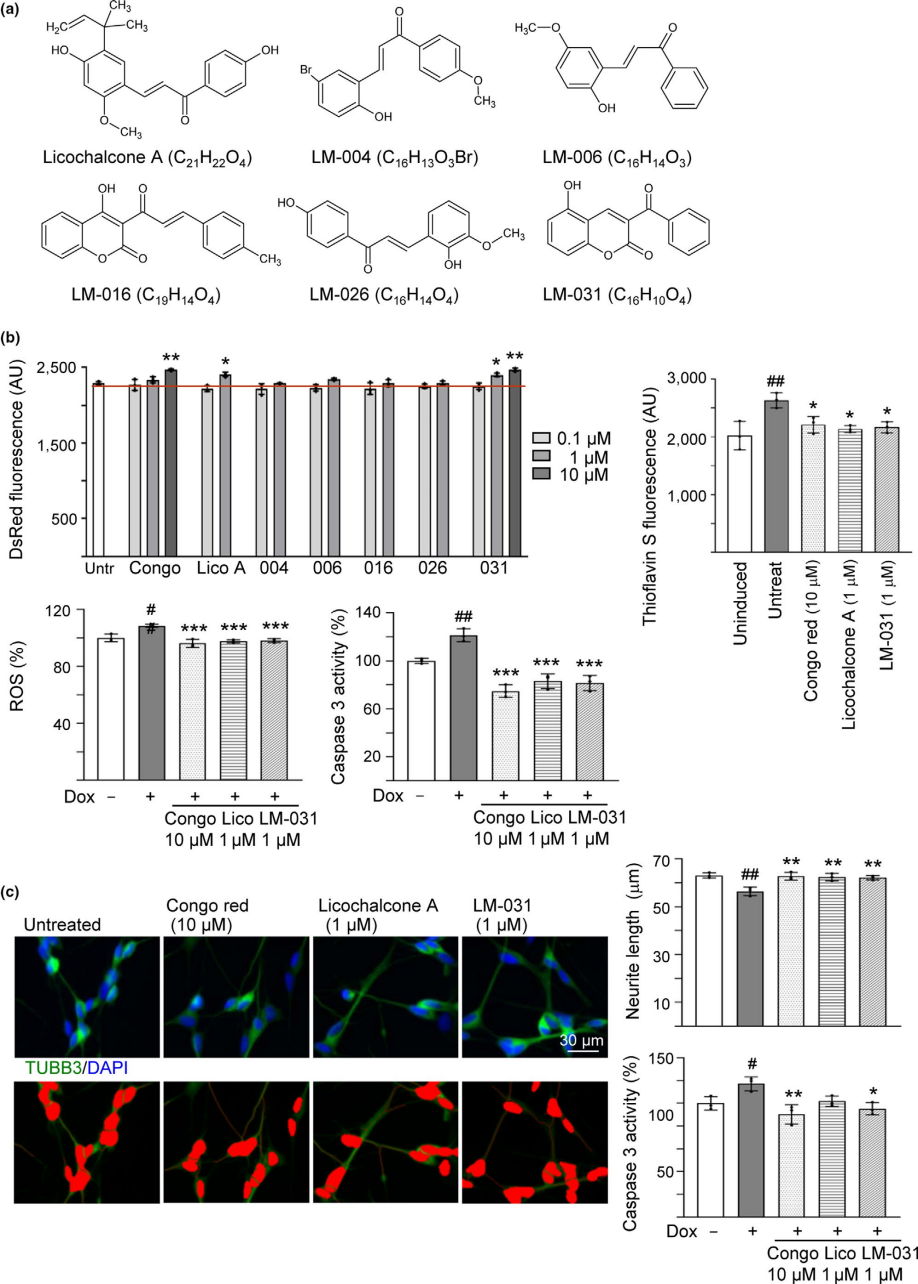
Effects of the test compounds on ΔK280 Tau_RD_‐DsRed 293 and SH‐SY5Y cells. (a) Structure, formula, and molecular weight of licochalcone A and LM compounds 004, 006, 016, 026, and 031. (b) Effects of the test compounds on inhibiting Tau misfolding, ROS production, and caspase 3 activity. ΔK280 Tau_RD_‐DsRed 293 cells were treated with congo red or test compounds (0.1–10 μM) for 3 days. DsRed fluorescence was measured in wells containing at least 80% viable cells (*n* = 3). In addition, thioflavin S fluorescence, ROS, and caspase 3 activity were measured on cells untreated or treated with congo red (10 µM), licochalcone A (1 µM), or LM‐031 (1 µM) (*n* = 3). The relative ROS and caspase 3 activity of uninduced cells were normalized (100%). (c) Neuroprotective effects of the test compounds. ΔK280 Tau_RD_‐DsRed SH‐SY5Y cells were treated with congo red (10 µM), licochalcone A (1 µM), or LM‐031 (1 µM) for 7 days, and caspase 3 activity assay was measured (*n* = 3), with uninduced cells normalized as 100%. TUBB3 (neuronal class III β‐tubulin) staining was performed to quantify the extent of neurite outgrowth (*n* = 3). Nuclei were counterstained with DAPI. Upper row, merged TUBB3 (green) and DAPI (blue) signals; lower row, images of the neurites and the body (red) outlined for outgrowth quantification. *p* values in (b) and (c): comparisons between induced and uninduced cells (^#^: *p* < .05 and ^##^: *p* < .01), or between treated and untreated cells (*: *p* < .05, **: *p* < .01, and ***: *p* < .001). (DsRed and Thioflavin S fluorescence: two‐tailed Student's *t* test; ROS, caspase 3 activity, and neurite outgrowth: one‐way ANOVA with a *post hoc* Tukey test)

Licochalcone A and five derivative LM compounds were tested (Figure 1a). All six compounds were soluble up to 100 μM in a cell culture medium (Figure S1a). Based on molecular weight (MW), hydrogen bond donors (HBD), hydrogen bond acceptors (HBA), and calculated octanol/water partition coefficient (cLogP), all six compounds meet Lipinski's criteria in predicting oral bioavailability (Lipinski, Lombardo, Dominy, & Feeney, 1997) (Figure S1b). With a polar surface area (PSA) less than 90 Å^2^, all six compounds were predicted to diffuse across the blood–brain barrier (BBB) (Hitchcock & Pennington, 2006), as also suggested by online BBB predictor (Figure S1b). To choose the optimal treatment dose used for the best efficacy of the candidate compounds, a specific type of biphasic dose response of a compound called hormesis should take into consideration. Evidence has shown that a wide range of pharmaceutical products display a hermetic dose‐response feature, characterized by a low‐dose stimulation and a high‐dose inhibition (Calabrese et al., 2018; Concetta Scuto et al., 2019; Pilipenko et al., 2019). Therefore, we tested the compounds at a range of doses to find the optimal dose for the best therapeutic effect and the least cytotoxicity. Firstly, MTT assay was performed using uninduced ∆K280 Tau_RD_‐DsRed 293 and retinoic acid‐differentiated SH‐SY5Y cells following treatment with the test compounds (0.1–10 μM) for 3 (for 293 cells) or 7 (for SH‐SY5Ycells) days. The IC_50_ values of congo red, licochalcone A, LM‐004, LM‐006, LM‐016, LM‐026, and LM‐031 in uninduced 293/SH‐SY5Y cells were 36.4/27.4, 7.0/5.8, 6.4/4.8, 6.4/4.7, 9.3/6.0, 7.7/5.4, and 55.9/6.8 μM, respectively (Figure S1c). Congo red is an amyloidogenic dye known to reduce Aβ neurotoxicity (Lorenzo & Yankner, 1994).

### 2.2 Reduction of Tau misfolding, ROS, and caspase 3 activity of licochalcone A and LM‐031 in ∆K280 Tau_RD_‐DsRed‐expressing 293 cells

In ∆K280 Tau_RD_‐DsRed 293 cells, a proaggregant (ΔK280) Tau repeat domain (Tau_RD_) containing residues 244–372 of the full‐length 4R2N Tau441 was fused in‐frame with DsRed fluorophore. Compared to the wild‐type Tau_RD_, the poorly folded ΔK280 Tau_RD_ formed aggregates, which adversely affected the folding of fused DsRed and thus decreased DsRed fluorescence, as also evidenced by increase in thioflavin S fluorescence emission (Chang et al., 2017). Thioflavin S is a reporter dye for studies of Tau toxicity and pharmacologic prevention in cell model of tauopathy (Pickhardt et al., 2017). It binds to amyloid fibrils but not monomers and gives a distinct increase in fluorescence emission. Inhibition of Tau aggregation may improve DsRed misfolding, leading to increased fluorescence in ΔK280 Tau_RD_‐DsRed‐expressing cells. Utilizing the established Tet‐on ΔK280 Tau_RD_‐DsRed 293 cells (Chang et al., 2016), licochalcone A and LM compounds were tested for effects of reducing Tau misfolding and anti‐oxidation (Figure S2a). Congo red was included for comparison. Based on the cell number analyzed, the IC_50_ cytotoxicity values of congo red, licochalcone A, LM‐004, LM‐006, LM‐016, LM‐026, and LM‐031 in 293 cells expressing ∆K280 Tau_RD_‐DsRed for 3 days were 36.9, 5.9, 5.0, 5.3, 9.4, 8.5, and 56.6 μM, respectively (Figure S2b). For all test compounds, IC_50_ cytotoxicity values in 293 cells without or with inducing Tau_RD_‐DsRed expression were similar, and LM‐031 has the least cytotoxicity (IC_50_ value of uninduced/induced cells: 55.9/56.6 μM) compared to other derivatives. In the following experiments, DsRed fluorescence was measured in wells containing at least 80% viable cells. As shown in Figure 1b, congo red at 10 µM significantly increased the ∆K280 Tau_RD_‐DsRed fluorescence compared to no treatment (108% versus 100%; *p* = .001). Significantly increased DsRed fluorescence was also observed with licochalcone A (105% for 1 µM treatment; *p* = .020) and LM‐031 (105%–108% for 1–10 µM treatment; *p* = .014–.004; dose‐response curve in Figure S2c) compared with untreated cells (100%). At 1 μM concentration, licochalcone A and LM‐031 effectively increased DsRed fluorescence and cell viability remained 94%–97% of cells without treatment, although cytotoxicity increased slightly for LM‐031 at 10 μM concentration in ∆K280 Tau_RD_‐DsRed‐expressing 293 cells (cell viability 91%). Due to the features of their effectiveness and less cytotoxicity, licochalcone A (1 μM) and LM‐031 (1 μM) were chosen as the candidate compounds for the following experiments. Thioflavin S fluorescence staining and quantification further revealed significantly increased thioflavin S fluorescence intensity in ΔK280 Tau_RD_‐DsRed‐expressing cells (130% versus 100%; *p* = .004), and treatment of congo red (10 µM), licochalcone A, or LM‐031 (1 µM) significantly decreased thioflavin S fluorescence intensity (109%–106% versus 130%; *p* = .037–.015) (Figure 1b). Representative DsRed and thioflavin S fluorescent images of ∆K280 Tau_RD_‐DsRed‐expressing cells untreated or treated with congo red (10 µM), licochalcone A (1 µM), or LM‐031 (1 µM) are shown in Figure S2d. Together, the results indicated that test compounds licochalcone A at 1 µM and LM‐031 at 1–10 µM reduced Tau misfolding in ΔK280 Tau_RD_‐DsRed‐expressing 293 cells.

Although known to reduce Aβ neurotoxicity (Lorenzo & Yankner, 1994), congo red was also reported as an agonist to drive aggregation within 293 cells expressing full‐length tau isoform htau40 (Bandyopadhyay, Li, Yin, & Kuret, 2007). Thus, we further examined the effects of congo red and tested chalcones on Tau misfolding using prokaryotic His‐tagged wild‐type (WT) and ∆K280 pET‐28a(+)‐Tau_RD_ proteins (Figure S3a) using thioflavin T binding assay and transmission electron microscopy (TEM) examination. When Tau_RD_ aggregate formation was measured with fluorescence generated by thioflavin T binding, increased aggregation was observed for both wild‐type (from 447 to 5,832 arbitrary unit (AU); *p* < .001) and ΔK280 (from 491 to 16,856 AU; *p* < .001) after 2 days’ incubation at 37°C. However, aggregation was significantly increased with ΔK280 as compared to wild‐type (16,856 versus 5,832 AU; *p* < .001), and ΔK280 Tau_RD_ aggregation was significantly reduced by congo red (from 16,856 to 4,774 AU; *p* < .001) and LM‐004 (from 16,856 to 6,758 AU; *p* = .002) at 10 µM concentration (Figure S3b). TEM examination of structures of Tau aggregates also revealed reduced Tau aggregates with congo red and LM‐004 treatment (10 µM) (Figure S3c). The results demonstrated that congo red and LM‐004 directly interfered with ΔK280 Tau_RD_ aggregate formation.

Misfolded Tau may increase the production of reactive oxygen species (ROS) (Cente, Filipcik, Pevalova, & Novak, 2006). To examine the anti‐oxidative effect of licochalcone A and LM‐031 on Tet‐On ∆K280 Tau_RD_‐DsRed 293 cells, ROS level was evaluated. As also shown in Figure 1b, pretreatment with congo red (10 µM), licochalcone A (1 µM), or LM‐031 (1 µM) significantly reversed the ROS level elevated by misfolded Tau production compared to no treatment (from 109% to 96%–98%; *p* < .001). These data showed the anti‐oxidative effect of licochalcone A and LM‐03l. In addition, pretreatment with congo red (10 µM), licochalcone A (1 µM), or LM‐031 (1 µM) significantly reversed the caspase 3 activity elevated by misfolded Tau production compared to no treatment (from 121% to 75%–83%; *p* < .001) (Figure 1b).

### 2.3 Promotion of neurite outgrowth and reduction of caspase 3 activity of licochalcone A and LM‐031 in ∆K280 Tau_RD_‐DsRed‐expressing SH‐SY5Y cells

Since licochalcone A or LM‐031 at 1 µM has good effects in anti‐oxidation and anti‐aggregation, the neuroprotective effects of licochalcone A or LM‐031 were evaluated using ∆K280 Tau_RD_‐DsRed‐expressing SH‐SY5Y cells (Figure S4a). As shown in Figure 1c, under the condition of retinoic acid (10 μM)‐induced neuronal differentiation, ∆K280 Tau_RD_‐DsRed overexpression significantly reduced the neurite length (from 63 to 56 μm; *p* = .002). Pretreatment with congo red (10 µM), licochalcone A (1 µM), or LM‐031 (1 µM) successfully rescued this impairment of neurite outgrowth (from 56 μm to 62–63 μm; *p* = .005–0.002). In addition, pretreatment with congo red (10 µM), licochalcone A, or LM‐031 (1 µM) reduced the caspase 3 activity compared to no treatment (from 117% to 90%–102%; *p* = .076–0.003).

### 2.4 Molecular targets of LM‐031 in ∆K280 Tau_RD_‐DsRed‐expressing SH‐SY5Y cells

**Figure 2:**
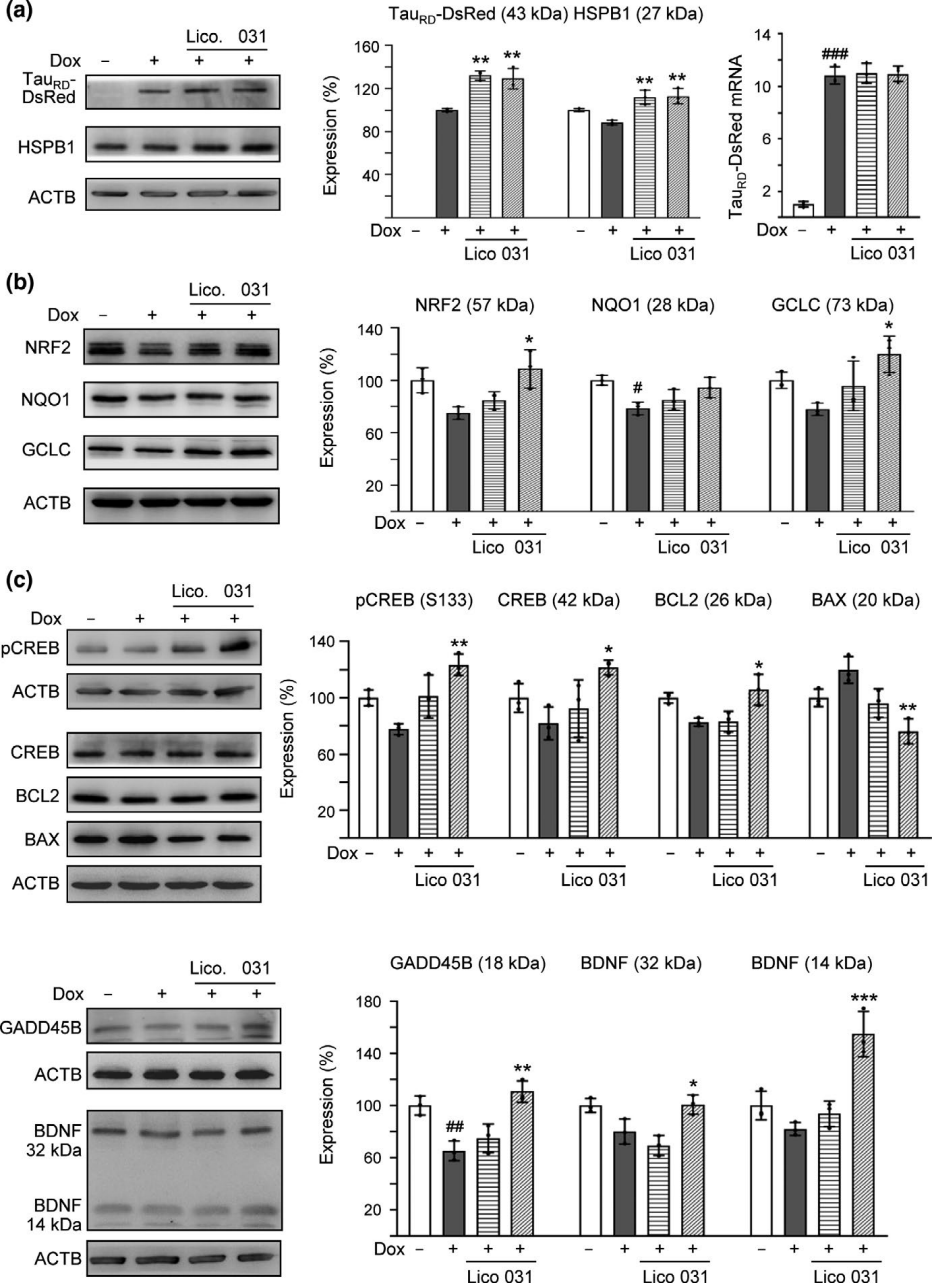
Enhanced expression of HSPB1, NRF2, and CREB pathways following LM‐031 administration in ΔK280 Tau_RD_‐DsRed SH‐SY5Y cells. Relative (a) HSPB1 and soluble Tau_RD_‐DsRed, (b) NRF2, GCLC, and NQO1, as well as (c) CREB, pCREB, BCL2, BAX, GADD45B, and BDNF protein levels were analyzed through immunoblotting using specific antibodies. Protein levels were normalized to β‐actin internal control. Relative protein levels are shown on the right side of the representative Western blot images. The relative protein level in uninduced cells was normalized (100%). For Tau_RD_‐DsRed, the soluble level in induced cells was set at 100%. In addition, ΔK280 Tau_RD_‐DsRed mRNA levels (a) were quantitated by real‐time PCR (*n* = 3). *p* values: comparisons between induced and uninduced cells (^#^: *p* < .05 and ^##^: *p* < .01), or between treated and untreated cells (*: *p* < .05, **: *p* < .01, and ***: *p* < .001) (*n* = 3). (one‐way ANOVA with a *post hoc* Tukey test)

The small molecular chaperone HSPB1 is involved in prevention of Tau misfolding to rescue hyperphosphorylated Tau‐mediated cell death (Shimura, Miura‐Shimura, & Kosik, 2004). Therefore, we examined whether licochalcone A and LM‐031 upregulate HSPB1 expression to assist Tau folding in ∆K280 Tau_RD_‐DsRed SH‐SY5Y cells (Figure S4a). Addition of licochalcone A or LM‐031 (1 µM) significantly increased HSPB1 expression (from 88% to 112%–113%; *p* = .002) and soluble ΔK280 Tau_RD_‐DsRed level (from 100% to 129%–132%; *p* = .003–.002), without affecting the relative ΔK280 Tau_RD_‐DsRed/HPRT1 RNA level (10.9‐ to 11.0‐fold of induction; *p* > .05) (Figure 2a).

In addition to HSPB1 chaperone, we examined the effects of licochalcone A and LM‐031 on expression levels of NRF2, CREB, and downstream targets (Andrisani, 1999; Zhang et al., 2013) in ∆K280 Tau_RD_‐DsRed‐expressing SH‐SY5Y cells (Figure S4a). Addition of LM‐031 (1 µM) increased NRF2 (from 75% to 109%; *p* = .012) and downstream NQO1 (NAD(P)H quinone dehydrogenase 1) (from 79% to 94%; *p* = .063) and GCLC (glutamate‐cysteine ligase catalytic subunit) (from 78% to 120%; *p* = .014) protein levels (Figure 2b). Furthermore, addition of LM‐031 (1 µM) significantly increased CREB (from 82% to 122%; *p* = .024), pCREB (S133) (from 78% to 124%; *p* = .001), downstream BDNF (brain‐derived neurotrophic factor) (32 kDa: from 83% to 101%; 14 kDa: from 82% to 155%; *p* = .043–<.001), BCL2 (BCL2 apoptosis regulator) (from 83% to 106%; *p* = .016), and GADD45B (growth arrest and DNA damage inducible beta) (from 65% to 111%; *p* = .001) protein levels (Figure 2c). In response to the anti‐apoptotic BCL2 change, addition of LM‐031 significantly reduced the expression of proapoptotic BAX (BCL2‐associated X, apoptosis regulator) (from 120% to 76%; *p* = .001).

BDNF binds to TRKB (neurotrophic receptor tyrosine kinase 2) to activate AKT (AKT serine/threonine kinase 1) and ERK (mitogen‐activated protein kinase 1) pathways, both of which play beneficial roles in AD (Nagahara et al., 2009). Therefore, we examined the expression levels of AKT, pAKT (S473), ERK1/2, and pERK1/2 (T202/Y204) following treating ∆K280 Tau_RD_‐DsRed‐expressing SH‐SY5Y cells with licochalcone A or LM‐031. As shown in Figure S4b, while the levels of AKT and ERK1/2 were not notably affected, the decreased pAKT (S473) (from 84% to 113%; *p* = .027) and pERK1/2 (T202/Y204) (from 90% to 117%; *p* < .001) expression levels were rescued with LM‐031 treatment.

### 2.5 NFR2 and CREB as therapeutic targets of LM‐031 in ∆K280 Tau_RD_‐DsRed‐expressing SH‐SY5Y cells

**Figure 3:**
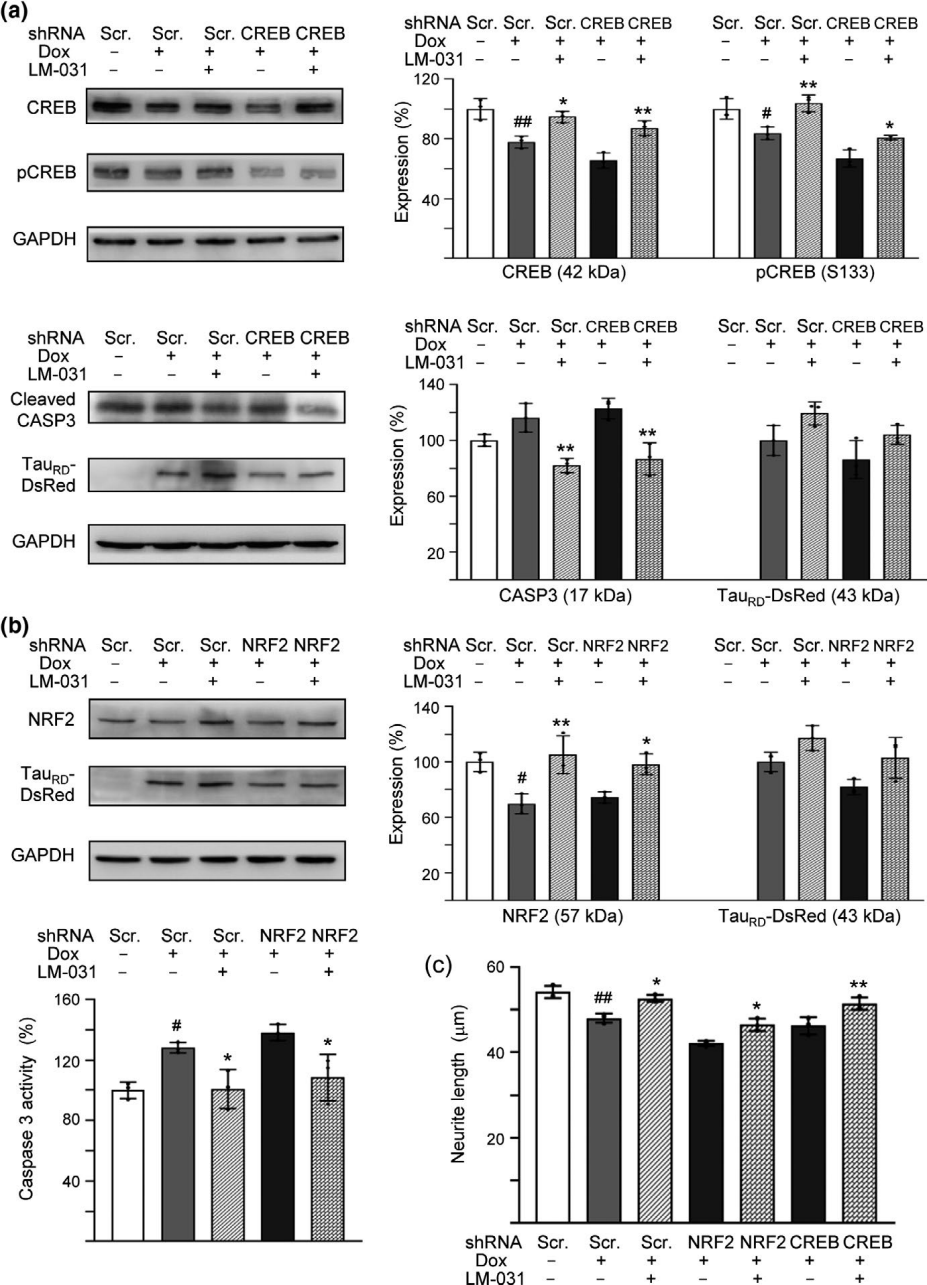
NRF2 and CREB as therapeutic targets in LM‐031‐treated ΔK280 Tau_RD_‐DsRed SH‐SY5Y cells. (a) Western blot analysis of CREB, pCREB, cleaved CASP3, and Tau_RD_‐DsRed levels in compound‐treated cells infected with CREB‐specific or scrambled shRNA‐expressing lentivirus. GAPDH was used as a loading control. To normalize, the relative CREB, pCREB, or CASP3 of uninduced cells, or relative Tau_RD_‐DsRed of untreated cells was set at 100%. (b) Western blot analysis of NRF2 and Tau_RD_‐DsRed levels (loading control: GAPDH) and caspase 3 activity assay in compound‐treated cells infected with NRF2‐specific or scrambled shRNA‐expressing lentivirus. To normalize, the relative NRF2 level or caspase 3 activity of uninduced cells, or relative Tau_RD_‐DsRed of untreated cells was set at 100%. (c) Neurite outgrowth quantification. *p* values: comparisons between induced and uninduced cells (^#^: *p* < .05 and ^##^: *p* < .01), or between compound‐treated and untreated cells (*: *p* < .05 and **: *p* < .01) (*n* = 3). (one‐way ANOVA with a *post hoc* Tukey test)

To validate the potential of NFR2 and CREB as therapeutic targets of LM‐031, we knocked down NFR2 and CREB expression through lentivirus‐mediated shRNA targeting in ∆K280 Tau_RD_‐DsRed‐expressing SH‐SY5Y cells (Figure S5a). Transduction of CREB or NRF2 shRNA attenuated the CREB mRNA expression from 81% to 67% (*p* = .069) or NRF2 mRNA expression from 71% to 60% (*p* = .151), although the effect of silencing was modest (Figure S5b). In scrambled shRNA‐infected cells, ∆K280 Tau_RD_‐DsRed overexpression significantly decreased NRF2 (70%; *p* = .010) and CREB, pCREB (S133) (78%–84%; *p* = .002–0.020) expression, while treatment with LM‐031 significantly increased NRF2 (from 70% to 105%; *p* = .003) and CREB, pCREB (from 78%–84% to 95%–104%; *p* = .016–0.005) levels (Figure 3a,b). LM‐031 addition also counteracted NRF2 (from 74% to 98%; *p* = .039) and CREB, pCREB (from 66%–67% to 81%–87%; *p* = .048–.003) reduction in NRF2 or CREB‐specific shRNA‐infected cells. The cleaved CASP3 level in scrambled or CREB‐specific shRNA‐infected cells was significantly reduced by LM‐031 treatment (from 116%–123% to 82%–87%; *p* = .003–.002) (Figure 3a). In line with NRF2 expression, caspase 3 activity in scrambled or NRF2‐specific shRNA‐infected cells was also significantly reduced by LM‐031 treatment (from 128%–138% to 101%–108%; *p* = .039–.024) (Figure 3b). Moreover, addition of LM‐031 improved ΔK280 Tau_RD_‐DsRed misfolding and enhanced soluble Tau_RD_‐DsRed protein level in scrambled (from 100% to 117%–119%, *p* = .209–.170), NRF2‐specific (from 82% to 103%, *p* = .106), or CREB‐specific (from 86% to 104%, *p* = .224) shRNA‐infected ΔK280 Tau_RD_‐DsRed SH‐SY5Y cells, although not significantly (Figure 3a, b).

Furthermore, ∆K280 Tau_RD_‐DsRed overexpression significantly reduced the neurite length in scrambled shRNA‐infected cells (from 54 to 48 µm; *p* = .002). Pretreatment with LM‐031 successfully rescued this neurite outgrowth impairment in scrambled (from 48 to 53 µm; *p* = .012), NRF2‐specific (from 42 to 46 µm; *p* = .016), or CREB‐specific (from 46 to 51 µm; *p* = .004) shRNA‐infected cells (Figure 3c, Figure S5c). These results suggested that LM‐031 exerted neuroprotective effects by upregulating NRF2 and CREB expression.

### 2.6 PAMPA to assess BBB permeability of LM‐031

To date, parallel artificial membrane permeability assay (PAMPA) models that exhibit a high degree of correlation with permeation across a variety of barriers, including BBB (Di, Kerns, Fan, McConnell, & Carter, 2003; Ottaviani, Martel, Escarala, Nicolle, & Carrupt, 2008), have been developed. We then used PAMPA‐BBB method to estimate the passive BBB permeability of LM‐031. Quality control compounds, testosterone (high‐permeability marker), theophylline (low‐permeability marker), and Lucifer yellow (integrity marker), were included for comparison. The effective permeability (P_e_) values of testosterone and theophylline were 21.75 and 0.11 (10^–6^ cm/s), respectively, representing high (>4 × 10^–6^ cm/s) and low (<2 × 10^–6^ cm/s) BBB permeable controls (Figure S6). The BBB permeability values of testosterone and theophylline were similar to the previously published results (Di et al., 2003). The transport of Lucifer yellow was undetectable, indicating good membrane integrity (below the 0.1% cut off). The P_e_ value of LM‐031 was 4.80 ± 0.12 (10^–6^ cm/s), suggesting that LM‐031 could be categorized as a high BBB permeable compound (P_e_ > 4 × 10^–6^ cm/s) in PAMPA‐BBB measurement.

### 2.7 LM‐031 ameliorated spatial learning and memory deficit in STZ‐treated 3 × Tg‐AD mice

**Figure 4:**
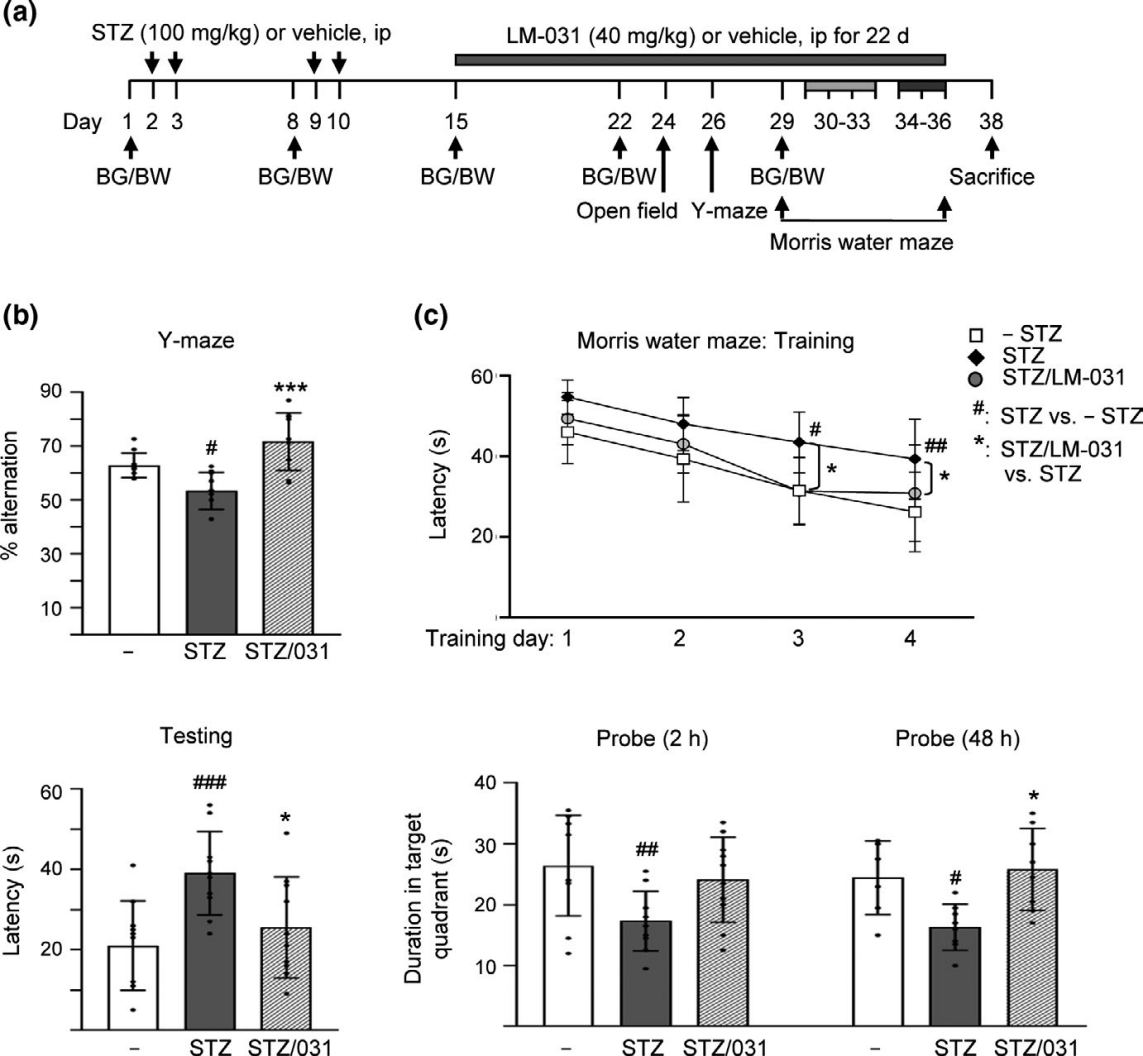
LM‐031 rescues spatial working memory, spatial learning, and memory in STZ‐treated 3 × Tg‐AD mice. (a) Experimental flow chart. Blood glucose (BG) and body weight (BW) were measured on days 1, 8, 15, 22, and 29. Mice were i.p. injected with streptozocin (STZ, 100 mg/kg) or vehicle (0.1 M sodium citrate pH4.5) on days 2, 3, 9, and 10, and then with LM‐031 (40 mg/kg) or vehicle (DMSO:Cremophor EL:0.9% saline = 1:2:7) from days 15 to 36. Open‐field, Y‐maze and Morris water maze tasks were performed on days 24, 26, and 29–36, respectively. Mice in– STZ, STZ, and STZ/LM‐031 groups received vehicle, STZ, and STZ + LM‐031, respectively, during the course of the experiment. (b) Y‐maze measurement of spontaneous alternation rate in 8 min of testing period. (c) Morris water maze testing of latency to find the hidden platform (latency) in training and testing and duration in target quadrant in probe trials (2 and 48 hr). *p* values, STZ versus – STZ mice or STZ/LM‐031 versus STZ mice. ^#^ and *: *p* < .05, ^##^ and **: *p* < .01, and ***: *p* < .001. (one‐way ANOVA with a *post hoc* Tukey test)

3 × Tg‐AD mice were used as an in vivo animal model to explore the potential of LM‐031 for AD treatment. At 6 months of age, the homozygous 3 × Tg‐AD mice displayed extracellular β‐amyloid plaques and intraneuronal Aβ buildup in the brain (Oddo et al., 2003), accompanied by more training to learn the Morris water maze task (Billings, Oddo, Green, McGaugh, & LaFerla, 2005). Intraneuronal aggregates of conformationally altered hyperphosphorylated Tau were not evident until the 3 × Tg‐AD mice reach about 12 months of age (Oddo et al., 2003). As impairment of brain glucose metabolism correlates well with the clinical symptoms in AD (Mosconi, 2005) and STZ exacerbated cognitive deficits in 3 × Tg‐AD mice (Chen, Liang, et al., 2014), STZ‐induced hyperglycemia was applied by i.p. injections of 4 STZ doses to 6‐month‐old 3 × Tg‐AD mice to accelerate the development of AD phenotypes (Figure 4a). Although females exhibit a stronger phenotype, males were used instead as estrogen heightened fear responses in a range of fear and anxiety‐provoking situations (e.g., open field) (Morgan & Pfaff, 2001). As shown in Figure S7a, blood glucose measured during the course remained relatively constant for the normoglycemic group (– STZ, 107–116 mg/dl). STZ injection increased blood glucose significantly, from 113 mg/dl (day 1) to 140 mg/dl (day 8; *p* = .031) and 220–314 mg/dl (days 15–29; *p* < .001) in STZ group. LM‐031 treatment significantly reduced blood glucose on days 22–29 (from 284–314 mg/dl to 197–183 mg/dl, *p* < .001) in STZ/LM‐031 group. Nevertheless, on days 15–29, the blood glucose levels in STZ/LM‐031 group remained significantly increased (183–215 mg/dl) as compared to the normoglycemic group (– STZ, 107–112 mg/dl) (*p* < .001). These results showed that STZ induced a hyperglycemic condition in 6‐month‐old 3 × Tg‐AD mice, which can be partially improved by LM‐031 treatment. No significant change of body weight was observed among the three groups.

An open‐field test was conducted on day 24 to explore the spontaneous motor activities and anxious mood in mice. No significant change was observed in distance traveled and inactive time of 3 × Tg‐AD mice with or without STZ or LM‐031 (Figure S7b). On day 26, Y‐maze alternation task based on the natural tendency of the mice to explore novel environment was performed to assess spatial working memory. As shown in Figure 4b, Y‐maze alternation rate was reduced in hyperglycemic (STZ) 3 × Tg‐AD mice as compared to normoglycemic group (– STZ) (53% versus 63%; *p* = .029), and LM‐031 treatment (STZ/LM‐031 group) significantly increased alternation rate (from 53% to 72%; *p* < .001). The results suggest enhanced working memory in LM‐031‐treated hyperglycemic 3 × Tg‐AD mice.

To assess the effect of LM‐031 on hippocampus‐dependent learning and memory retrieval, we conducted Morris water maze task in mice with training (days 30–33), testing (day 34), and probe (days 34 and 36) trials (Figure 4c). After receiving one day's pretraining, the time spent to reach the hidden platform (escape latency time) improved for the following training days. When the performances of hyperglycemic (STZ) and normoglycemic (– STZ) 3 × Tg‐AD mice were compared, hyperglycemic mice spent more time searching for the platform on training day 3 (43 s versus 31 s; *p* = .028) and day 4 (40 s versus 26 s; *p* = .006) as compared with the normoglycemic mice. LM‐031 treatment reduced the latency of hyperglycemic mice on training day 3 (from 43 to 31 s; *p* = .017) and day 4 (from 40 to 30 s; *p* = .049). In the following testing on day 34, the latency in the hyperglycemic mice was significantly longer than that of the normoglycemic mice (39 s versus 21 s; *p* < .001) and LM‐031 treatment significantly reduced the latency (26 s versus 39 s; *p* = .037). Our results demonstrated that deficit in the spatial learning ability of 3 × Tg‐AD mice was enhanced with STZ‐induced hyperglycemia and the impairment of acquisition of spatial reference learning was mitigated with LM‐031 treatment. In the probe trials on days 34 and 36, the duration in target quadrant of the hyperglycemic mice was markedly less than that of the normoglycemic mice (17 s versus 26 s; *p* = .011–0.006), and LM‐031 treatment extended the time spent in the target quadrant (from 17 to 24 s; *p* = .060–0.047) for both 2 and 48 hr trials. Thus, LM‐031 treatment increased the retrieval of spatial memory in hyperglycemic 3 × Tg‐AD mice.

### 2.8 LM‐031 increased NeuN level and decreased Aβ and Tau levels in STZ‐treated 3 × Tg‐AD mice

**Figure 5:**
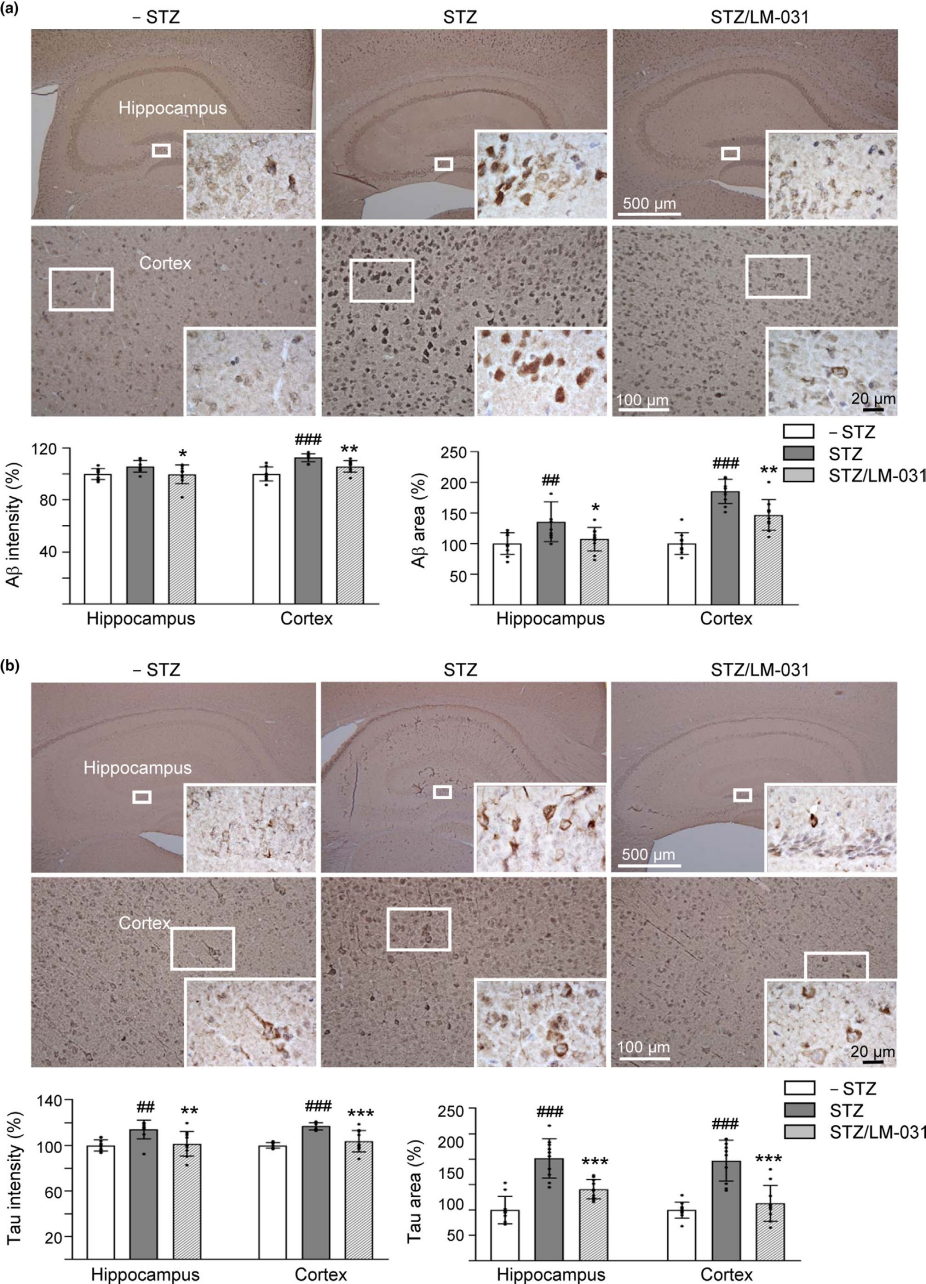
LM‐031 decreases Aβ and Tau immunoreactivity in STZ‐treated 3 × Tg‐AD mice. Mice in – STZ, STZ, and STZ/LM‐031 groups received vehicle, STZ, and STZ + LM‐031, respectively, during the course of the experiment. Representative IHC images for Aβ (a) and Tau (b), and intensity and area quantification in the hippocampus and cortex of mice. *p* values, STZ versus –STZ mice or STZ/LM‐031 versus STZ mice. ^#^: *p* < .05, ^##^ and **: *p* < .01, and ^###^ and ***: *p* < .001. (one‐way ANOVA with a *post hoc* Tukey test)

In addition to cognitive function, we examined NeuN (RNA binding protein, fox‐1 homolog (*C. elegans*) 3), Aβ, and Tau levels in 3 × Tg‐AD mice with or without STZ or LM‐031 treatment. Immunohistochemically, STZ treatment reduced NeuN level in dentate gyrus (DG, 90%, *p* = .031) and *Cornu Ammonis* areas 1 (CA1, 89%, *p* = .015) and 3 (CA3, 91%, *p* = .023) of the hippocampus of 3 × Tg‐AD mice (STZ group), whereas LM‐031 treatment could reduce this decrease to 97% (DG, *p* = .045), 96% (CA1, *p* = .148), and 95% (CA3, *p* = .053) (STZ/LM‐031 group) compared to the normoglycemic control (– STZ group, 100%) (Figure S7c). In addition, STZ treatment increased Aβ level (intensity: 106%–113%, *p* = .059–<.001; area: 136%–185%, *p* = .007–<.001) in the hippocampus and cortex of 3 × Tg‐AD mice (STZ group), whereas LM‐031 treatment decreased the level of Aβ (intensity: 100%–106%, *p* = .046–0.006; area: 107%–147%, *p* = .035–0.001) (STZ/LM‐031 group) compared to the normoglycemic control (– STZ group, 100%) (Figure 5a). Furthermore, STZ treatment increased Tau level (intensity: 114%–117%, *p* = .002–<.001; area: 202%–197%, *p* < .001) in the hippocampus and cortex of 3 × Tg‐AD mice (STZ group), whereas LM‐031 treatment could decrease the level of Tau (intensity: 101%–104%, *p* = .006–<0.001; area: 141%–113%, *p* < .001) (STZ/LM‐031 group) compared to the normoglycemic control (– STZ group, 100%) (Figure 5b). TEM examination of sarkosyl‐insoluble Tau also revealed reduced Tau aggregates in STZ/LM‐031 mice (Figure S7d). Although Tau pathology is thought to be downstream (and occur after) of amyloid pathology in 3 × Tg‐AD mice, the decrease of pathological Aβ aggregation may be explained by the anti‐aggregation, antioxidant, and neuroprotective effects of LM‐031 against Aβ misfolding and toxicity (Lee et al., 2018). Together, our findings confirmed neuronal loss and abnormal elevation of Aβ and Tau in the hippocampus and cortex of hyperglycemic 3 × Tg‐AD mice and LM‐031 treatment could rescue these pathologic changes.

### 2.9 LM‐031 augments NFR2 and pCREB expression in STZ‐treated 3 × Tg‐AD mice

**Figure 6:**
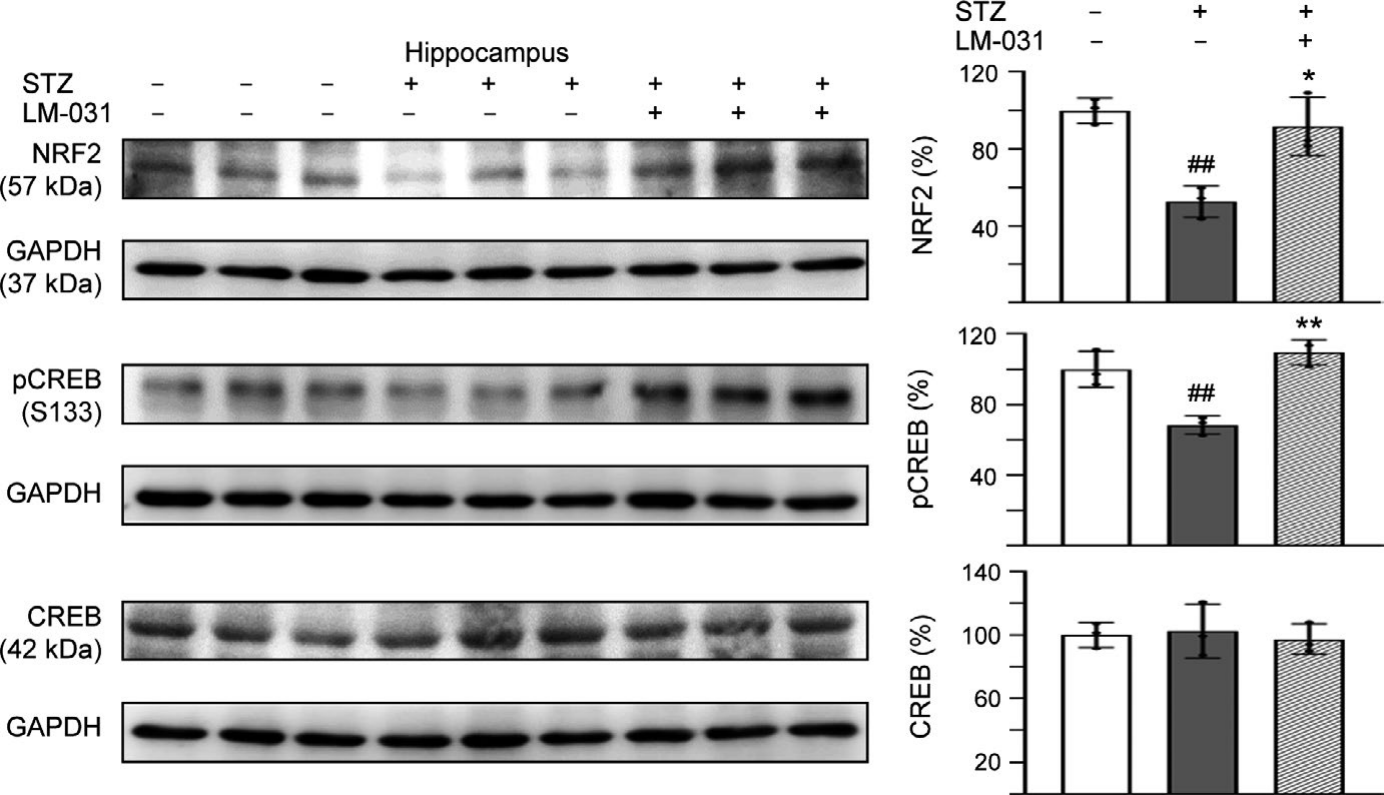
LM‐031 augments NFR2 and pCREB expression in STZ‐treated 3 × Tg‐AD mice. Expression levels of NRF2, pCREB, and CREB in hippocampus were analyzed by Western blot using GAPDH as a loading control. To normalize, the relative NRF2, pCREB, and CREB of, STZ mice was set as 100%. *p* values, STZ versus – STZ mice or STZ/LM‐031 versus STZ mice. *: *p* < .05, and ^##^ and **: *p* < .01. (one‐way ANOVA with a *post hoc* Tukey test)

Finally, we examined the effects of LM‐031 on alternations of NRF2 and CREB, pCREB levels in STZ‐treated 3 × Tg‐AD mice (Figure 6). STZ administration reduced NRF2 (53%, *p* = .004) and pCREB (68%, *p* = .006) levels in the hippocampus of 3 × Tg‐AD mice (STZ group), whereas LM‐031 treatment could reverse this reduction to 92% (NRF2, *p* = .010) or 110% (pCREB, *p* = .002) (STZ/LM‐031 group) compared to the normoglycemic control (– STZ group, 100%). However, the relative total CREB level was not significantly different among the three groups.

## 3 DISCUSSION

In this study, we demonstrated Tau misfolding reduction, anti‐oxidation, and neuroprotection effects of in‐house synthetic compound LM‐031. Treatment with LM‐031 increases soluble ∆K280 Tau_RD_‐DsRed protein and fluorescence intensity without changing the Tau_RD_‐DsRed RNA level (Figures 1b, 2a), which reflects that the increased fluorescence intensity is mainly due to the improvement of Tau misfolding state but not caused by increased Tau_RD_‐DsRed transcription. In other words, neurite outgrowth‐promoting effect of LM‐031 in our tauopathy model is probably due to the amelioration of tau misfolding, which leads to reduction of oxidative stress and caspase 3 activity (Figure 1). Further target identification from mechanistic studies indicates that LM‐031 upregulates HSPB1, NRF2, and CREB expression in differentiated ΔK280 Tau_RD_‐DsRed SH‐SY5Y cells to suppress apoptosis and promote neuron survival (Figures 2, 3).

HSPB1 has a potent protective effect against Aβ and Tau‐induced toxicity through preventing protein misfolding and aggregation (Chang et al., 2017; Lee et al., 2018). Overexpression of HSPB1 ameliorated symptoms of AD in APP/PS1 mice (Tóth et al., 2013). The different indolylquinoline (Chang et al., 2017) and chalcone (Lee et al., 2018 and this study) compounds with similar effect on enhancing HSPB1 expression are not uncommon, as compounds may bind to different promotor sites to enhance HSPB1 expression. We also proposed another hypothesis that one of them may act on upstream gene that controls the HSPB1 expression and the other directly on HSPB1.

Oxidative stress has been recognized as a contributing factor to aging and progression of multiple neurodegenerative diseases including AD. Increased production of ROS and reduced antioxidant defense could affect synaptic activity leading to cognitive dysfunction, and accumulation of hyperphosphorylated Tau protein could exacerbate ROS production (Tönnies & Trushina, 2017). The levels of HSPs correlate inversely with the levels of granular Tau oligomers in human brain, implicating that heat shock proteins may regulate soluble Tau protein levels to counteract the formation of granular Tau oligomers (Sahara et al., 2007). By activating HSPB1 expression to reduce aggregation, ROS and cytotoxicity in ΔK280 Tau_RD_‐DsRed‐expressing cells (Figure 1), LM‐031 could be a promising disease‐modifying compound for the treatment of AD and other oxidative stress‐related neurodegenerative diseases.

Among the mechanisms of protection against oxidative stress, activation of NRF2 increases levels of phase 2 antioxidant enzymes such as NQO1 and GCLC (Zhang et al., 2013). A significant decrease in nuclear NRF2 levels observed in postmortem brains of human AD cases suggests that NRF2‐mediated transcription is not activated in neurons in AD despite the presence of oxidative stress (Ramsey et al., 2007). Oxidative stress involving changes in NRF2 constitutes one of the early events in 3 × Tg‐AD mice (Mota et al., 2015). By combining the NRF2 activator 18α‐glycyrrhetinic acid and the NADH precursor nicotinamide, neuronal survival in 3 × Tg‐AD against β‐amyloid stress was additively increased (Ghosh, LeVault, & Brewer, 2014). In addition, carnosic acid, a compound that is activated by oxidative stress to stimulate NRF2 transcription pathway, exhibits neuroprotective effects both in vitro and in transgenic AD mice (Lipton et al., 2016). In our study, treatment with LM‐031 upregulated antioxidant NRF2 pathway and reduced proapoptotic BAX and CASP3 expression in pro‐aggregated Tau_RD_‐DsRed‐expressing SH‐SY5Y cells, supporting the role of LM‐031 in treating AD by preventing oxidative stress and blocking apoptosis pathway.

Synaptic dysfunction in AD has been a recent focus as memory formation is accompanied by altered synaptic strength and synaptic changes are highly correlated with the severity of clinical dementia (Teich et al., 2015). It is well known that CREB plays a crucial role in memory consolidation (Silva, Kogan, Frankland, & Kida, 1998), as phosphorylated CREB binds to CBP (CREB‐binding protein, a histone acetyltransferase that catalyzes histone acetylation), causing an increase in the transcription of memory‐associated genes (Kandel, 2004; Korzus, Rosenfeld, & Mayford, 2004). CREB downregulation has been implicated in the pathology of AD (Bartolotti, Bennett, & Lazarov, 2016; Pugazhenthi, Wang, Pham, Sze, & Eckman, 2011; Yamamoto‐Sasaki, Ozawa, Saito, Rosler, & Riederer, 1999). Overexpression of human wild‐type full‐length Tau in hippocampal neurons impaired synapse impairment and memory deficits by dephosphorylation of CREB mediated through upregulating calcium/calmodulin‐dependent protein phosphatase calcineurin and suppressing calcium/calmodulin‐dependent protein kinase IV (Yin et al., 2016). Thus, increasing the expression of CREB could be considered as a possible therapeutic target for tauopathies including AD. In our study, LM‐031 demonstrated its potential of enhancing CREB‐dependent gene transcription including BDNF, ERK, AKT, BCL2, and GADD45B, all of which are important to neuronal survival, in pro‐aggregated Tau_RD_‐DsRed‐expressing SH‐SY5Y cells (Figure 2), further highlighting its possible role as a novel compound targeting multiple pathways in AD treatment.

In this study, the therapeutic potential of LM‐031 in AD was further examined in 3 × Tg‐AD mice which displayed the pathology and synaptic deficits of AD in an age‐dependent manner (Oddo et al., 2003). AD occurs as a result of complex interactions between genes and risk factors (Panpalli Ates, Karaman, Guntekin, & Ergun, 2016). Substantial evidence has suggested that hyperglycemia provokes disease progression in mild cognitive impairment (Morris, Vidoni, Honea, Burns, & Alzheimer’s Disease Neuroimaging Initiative, 2014) and senile plaque formation in APP/PS1 transgenic mice (Wang et al., 2014). Therefore, hyperglycemia was used in this study to accelerate the phenotypes of 3 × Tg‐AD mice to assess therapeutic efficacy of LM‐031. In vivo, the observed beneficial effects of LM‐031 on learning and memory improvement in hyperglycemic 3 × Tg‐AD mice (Figure 4) could be mediated by upregulating NRF2 and pCREB and reducing Aβ and Tau levels in hippocampus and cortex of the hyperglycemic 3 × Tg‐AD mice (Figures 5, 6).

Both Aβ and ∆K280 Tau_RD_ attenuate the expression level of CREB in SH‐SY5Y cells (Lee et al., 2018 and Figure 2c). However, no apparent reduction of CREB protein level was observed in STZ‐induced hyperglycemic 3 × Tg‐AD mice (Figure 6). Although a previous study of brain gene expression of 3 × Tg‐AD mice revealed the reduced expression of CREB, the reduction did not reach statistical significance (Chen et al., 2012). CBP gene transfer to 3 × Tg‐AD mice increased the reduced pCREB to increase BDNF levels and ameliorate learning and memory deficits without affecting total CREB level (Caccamo, Maldonado, Bokov, Majumder, & Oddo, 2010). In intracerebroventricular STZ‐induced memory‐impaired rats, STZ injection significantly downregulated the pCREB but not CREB expression in the cerebral cortex and hippocampus (Rajasekar, Nath, Hanif, & Shukla, 2017). In STZ‐induced diabetic mice, pCREB/CREB and the expression of BDNF protein in the hippocampus were obviously decreased in STZ group, without affecting CREB level (Tang et al., 2018). In accordance with the previous studies using AD or STZ‐induced animal models, our study results suggest that the main pathology is reduced pCREB rather than total CREB and the compound enhancing pCREB is able to rescue the phenotype and neurodegeneration of STZ‐accelerated AD mice.

Currently, no effective treatment exists for modifying or preventing AD progression; thus, new strategies are desperately needed. Tau misfolding and aggregation cause oxidative stress and associated neuronal injury and cell death. Taken together, in‐house synthetic LM‐031 exerted neuroprotective effects in ΔK280 Tau_RD_‐DsRed SH‐SY5Y cells by upregulating molecular chaperone HSPB1 and NRF2/NQO1/GCLC to reduce oxidative stress and by activating CREB‐dependent BDNF/AKT/ERK and BCL2 for cell survival and anti‐apoptosis. With low cytotoxicity in human cells, high solubility in cell culture medium, high BBB permeability, and prediction of orally bioavailable, LM‐031 has potential for being developed as an AD therapeutic. Additional preclinical studies are required before applying this multi‐target compound to modify the progression of AD in clinical trials.

## 4 MATERIALS AND METHODS

In‐house LM compounds were synthesized and characterized by NMR spectrum. Tet‐On ∆K280 Tau_RD_‐DsRed 293 and SH‐SY5Y cells were cultured in Dulbecco's modified Eagles medium (DMEM) (293) or DMEM‐F12 (SH‐SY5Y) containing 10% fetal bovine serum, 5 μg/ml of blasticidin, and 100 μg/ml of hygromycin. ∆K280 Tau_RD_‐DsRed cell fluorescence assay, reactive oxygen species (ROS) assay, caspase 3 activity measurement, neurite outgrowth analysis, RNA interference, and Western blot analysis were performed.

For the in vivo LM‐031 treatment experiments, 6‐month‐old male homozygous 3 × Tg‐AD mice (*n* = 10 per group) were used. Mice were randomly divided into three groups: – streptozocin (STZ), STZ, and STZ/LM‐031. STZ‐induced hyperglycemia was used to accelerate the development of AD phenotype. Mouse body weight and blood glucose level were measured on days 1, 8, 15, 22, and 29. LM‐031 (40 mg/kg) or vehicle (DMSO:Cremophor EL:0.9% saline = 1:2:7) was intraperitoneally injected every day for 22 days from days 15 to 36. All animal procedures were ethically approved by the Institutional Animal Care and Use Committee of National Taiwan Normal University, Taipei, Taiwan (Permit Number: 103002) and were conducted in compliance with the ARRIVE (Animal Research: Reporting In Vivo Experiments) guidelines. Open‐field task, Y‐maze task, Morris water maze task, and immunohistochemistry and image analysis were performed.

Full methods are available in the Supporting Information.

## CONFLICT OF INTEREST

None declared.

## AUTHOR CONTRIBUTIONS

GJLC and CMC designed the study, analyzed data, and wrote the manuscript. THL and YJC performed most of the experimental work and analyzed data. CHL, CYL, CYC, YCC, and SMY performed some experiments. WL, HMHL, YRW, and KHC reviewed the manuscript. All authors approved the final version of the manuscript.

## Supporting information

Fig S1

Fig S2

Fig S3

Fig S4

Fig S5

Fig S6

Fig S7a‐c

Fig S7d

Supplementary Material

## Data Availability

The data that support the findings of this study are available in Mendeley Data at https://data.mendeley.com/datasets/f78fjp74c2/draft?a=c4977b15‐e804‐40de‐8425‐fa9c29594a26.
