# Supplementary figures and images for: Exploration of multi‐target effects of 3‐benzoyl‐5‐hydroxychromen‐2‐one in Alzheimer’s disease cell and mouse models
Source: Aging Cell. 2020 Jun 4;19(7):e13169. doi: 10.1111/acel.13169 (PMC7433010; doi:10.1111/acel.13169)

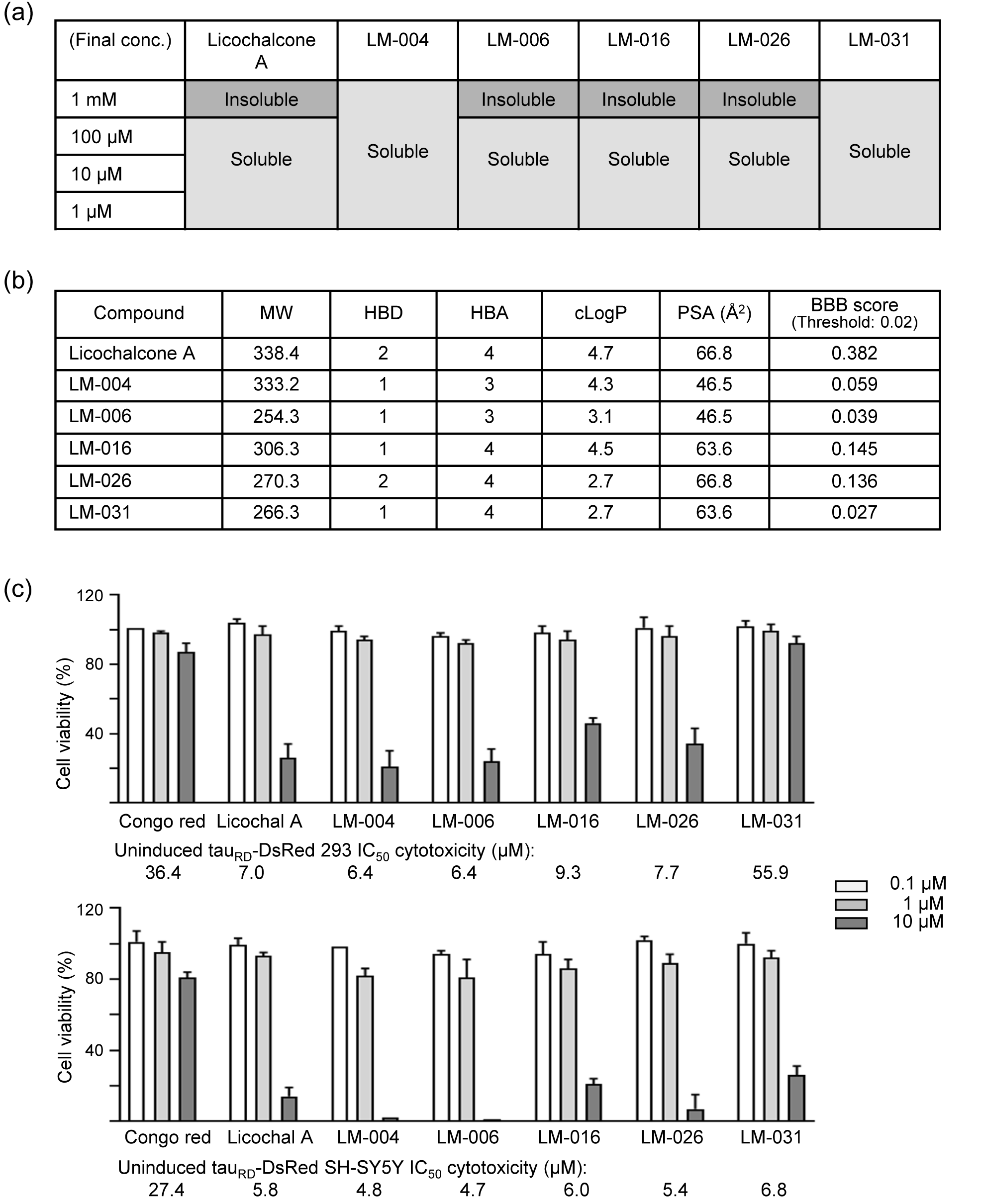

Supplement: Supplementary file 1 — Fig S1 [file ACEL-19-e13169-s001.tif]

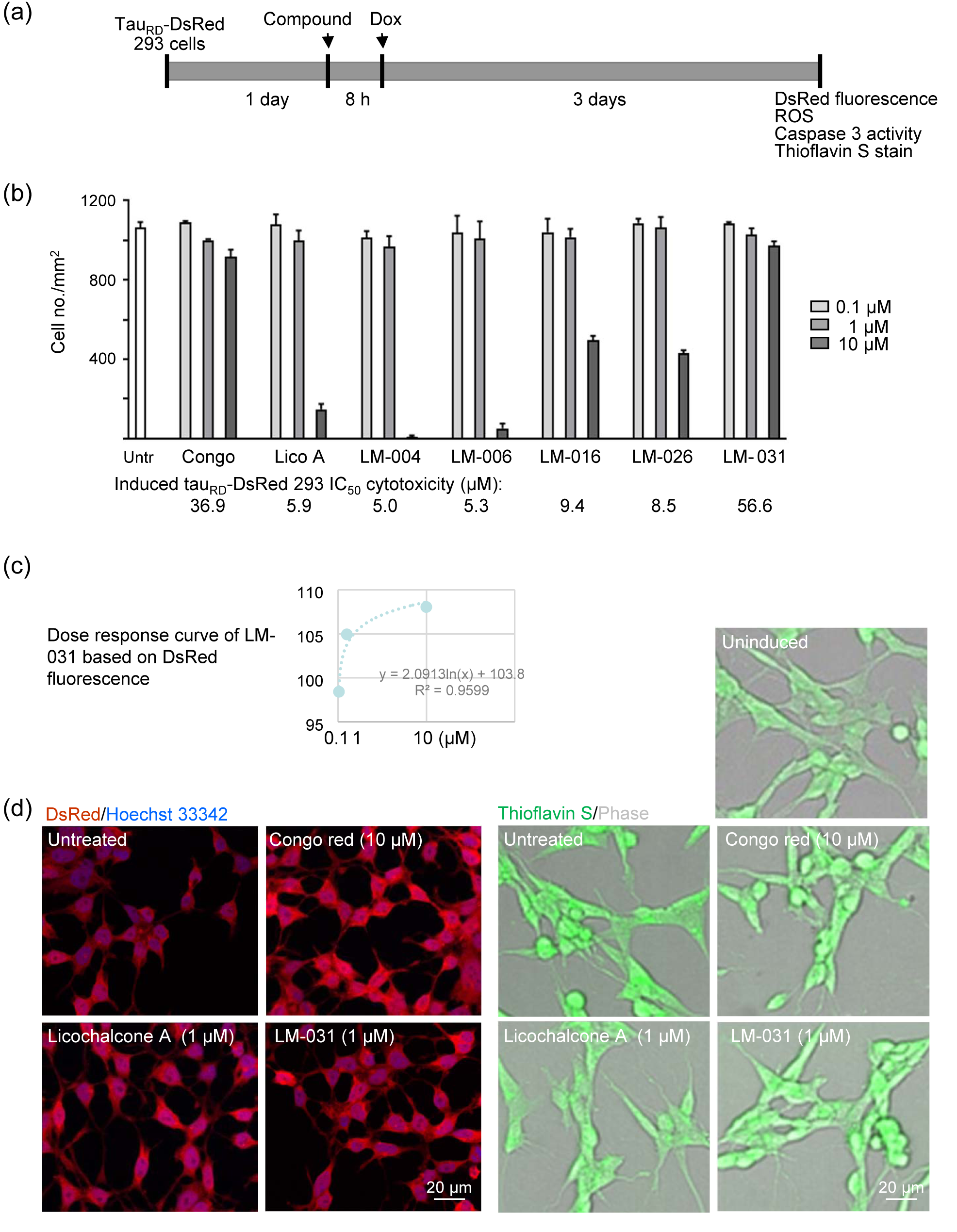

Supplement: Supplementary file 2 — Fig S2 [file ACEL-19-e13169-s002.tif]

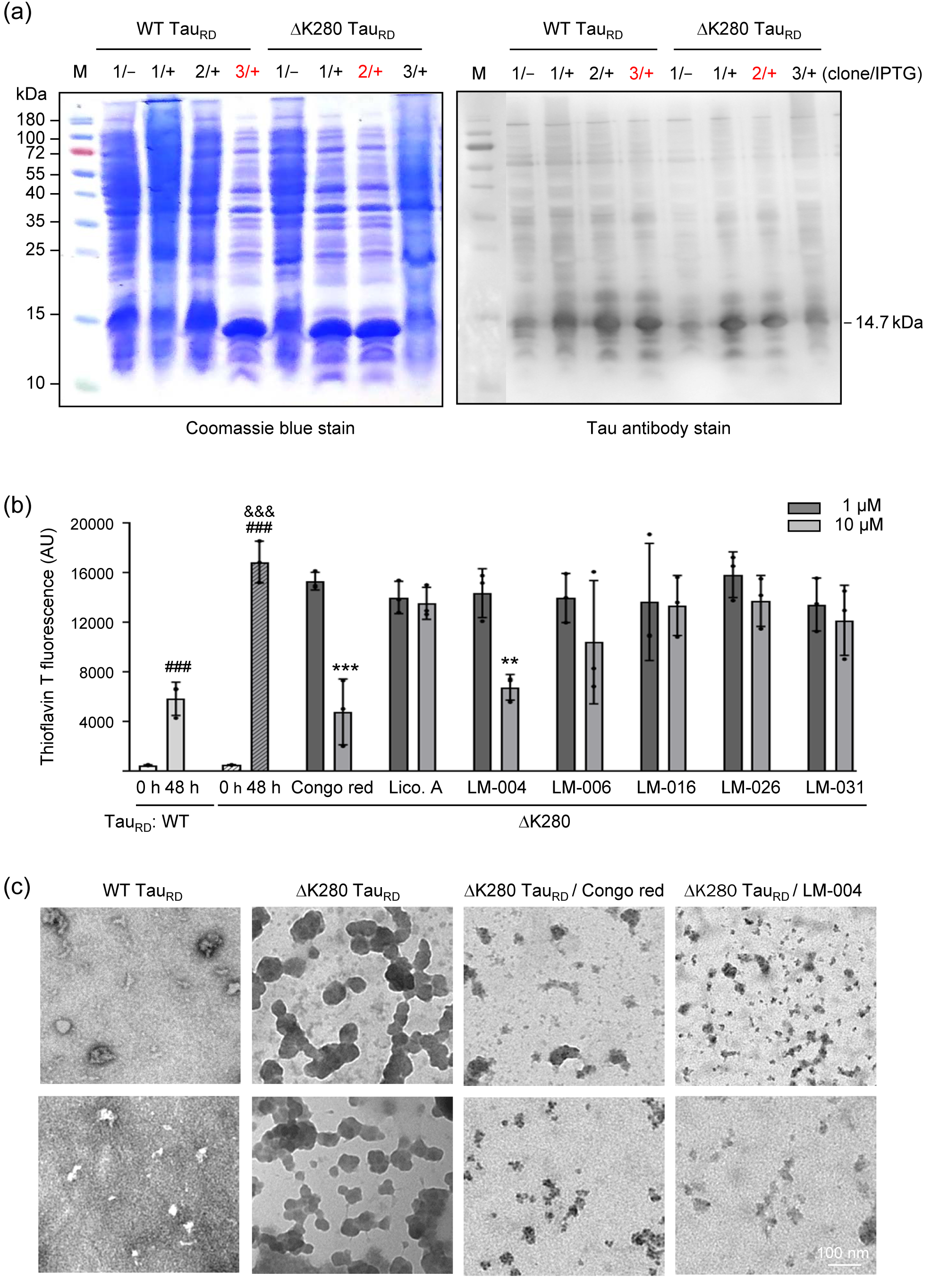

Supplement: Supplementary file 3 — Fig S3 [file ACEL-19-e13169-s003.tif]

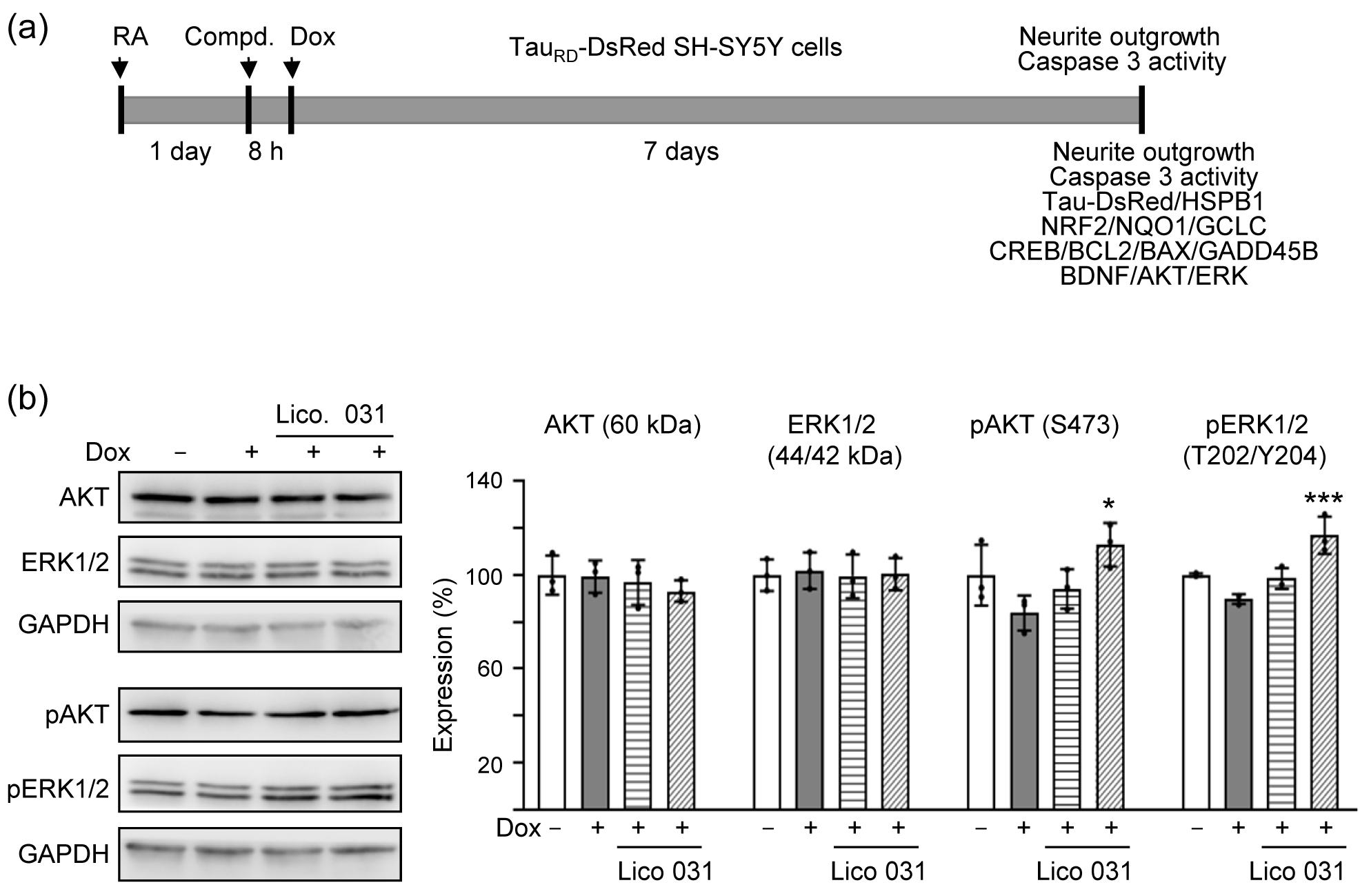

Supplement: Supplementary file 4 — Fig S4 [file ACEL-19-e13169-s004.tif]

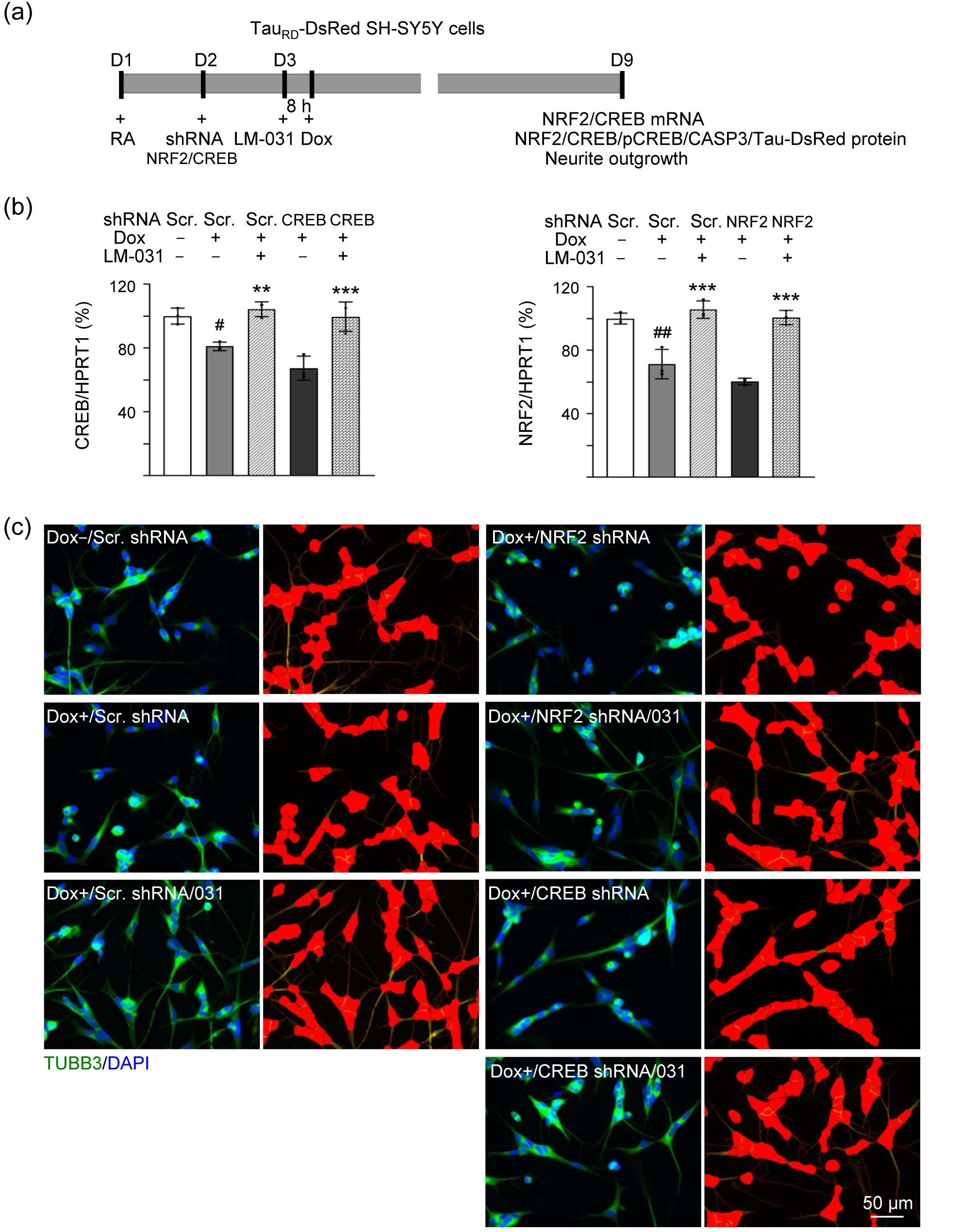

Supplement: Supplementary file 5 — Fig S5 [file ACEL-19-e13169-s005.tif]

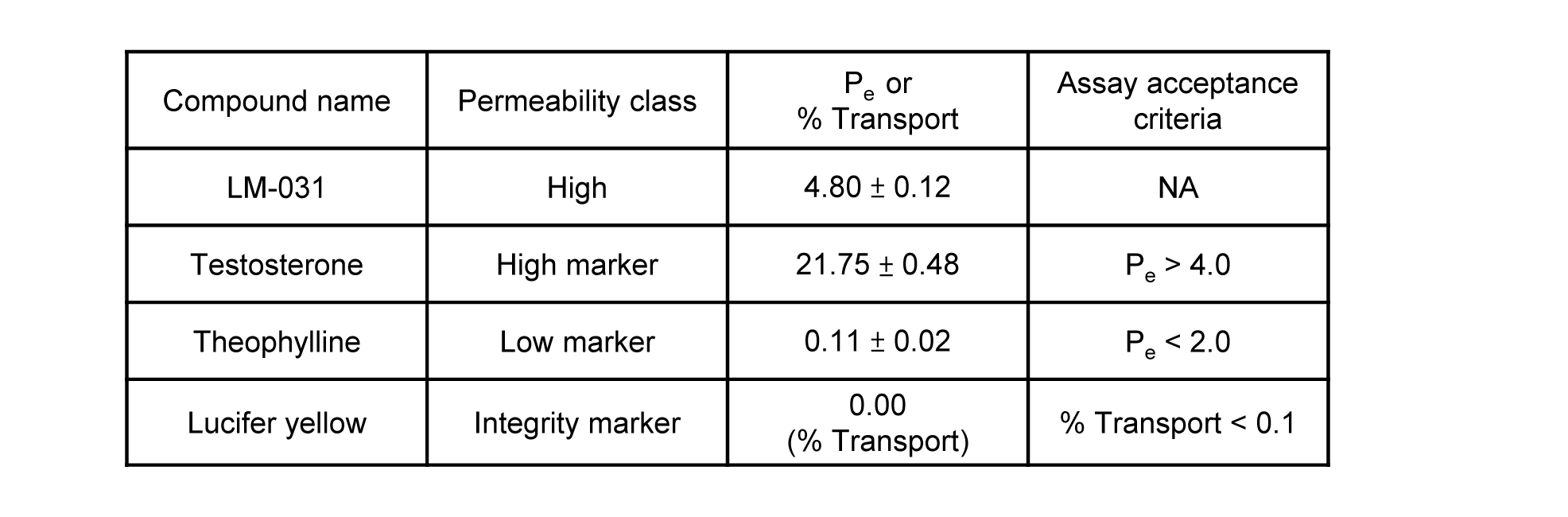

Supplement: Supplementary file 6 — Fig S6 [file ACEL-19-e13169-s006.tif]

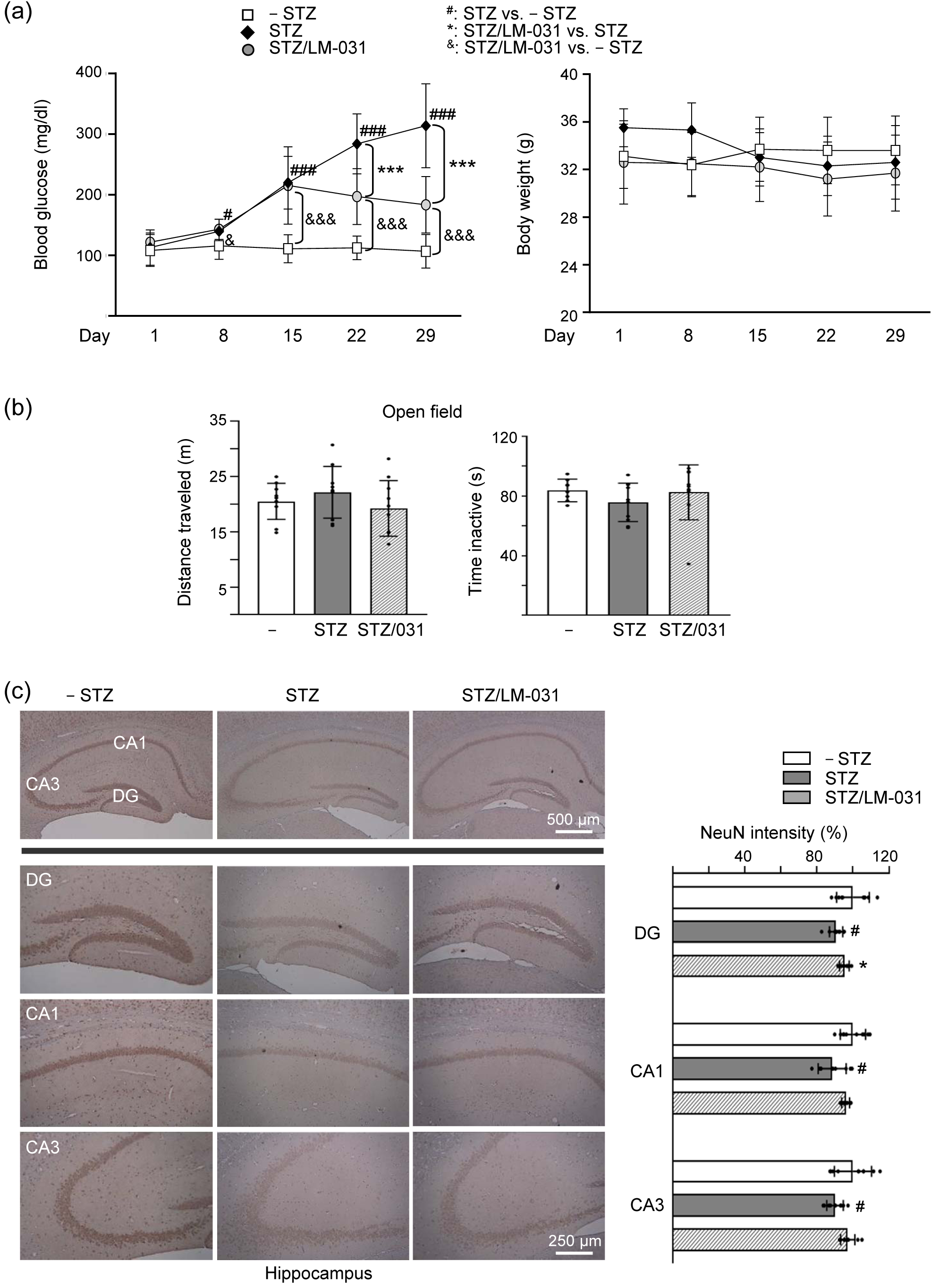

Supplement: Supplementary file 7 — Fig S7a‐c [file ACEL-19-e13169-s007.tif]

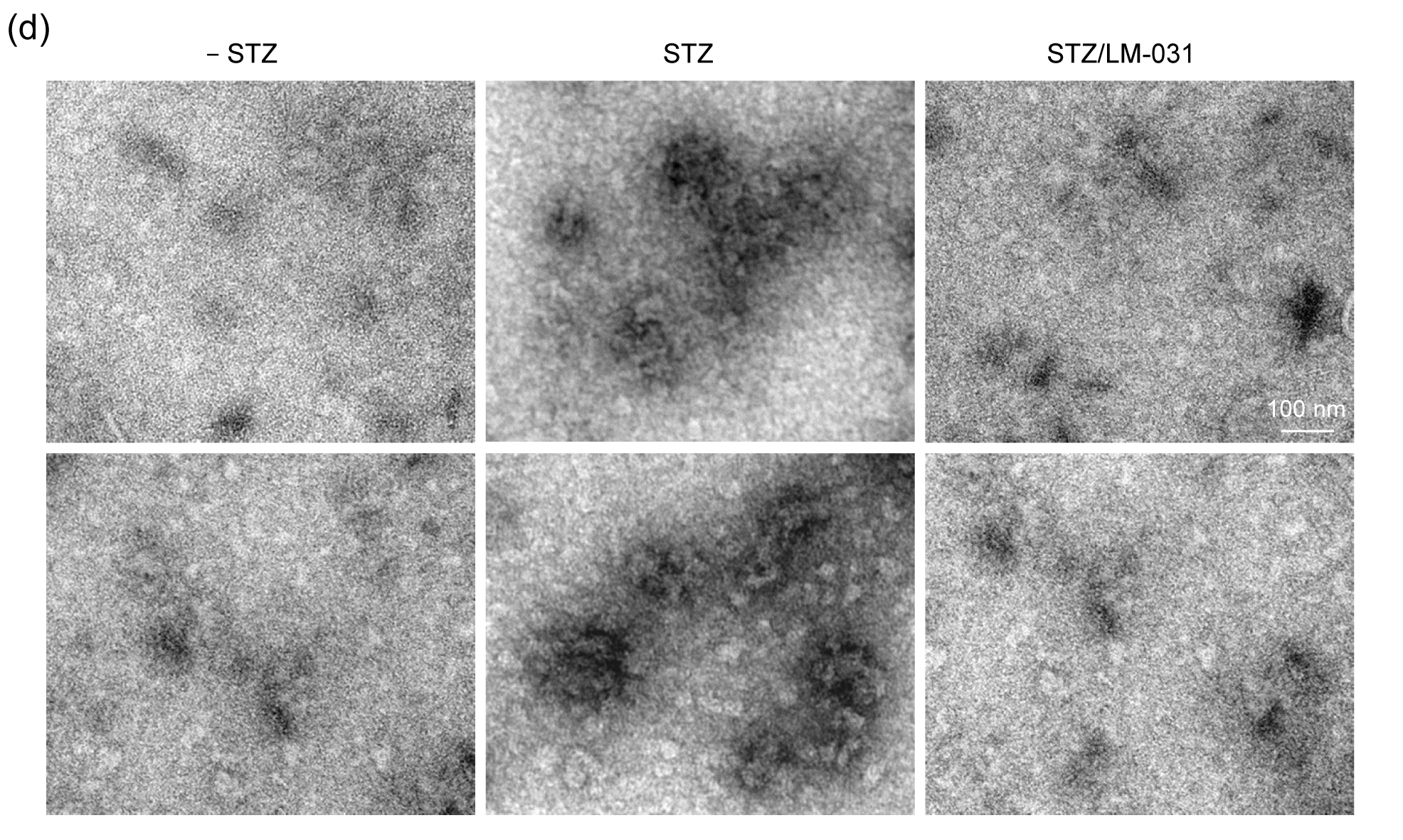

Supplement: Supplementary file 8 — Fig S7d [file ACEL-19-e13169-s008.tif]
