# Supplementary material for: Exploration of multi‐target effects of 3‐benzoyl‐5‐hydroxychromen‐2‐one in Alzheimer’s disease cell and mouse models
Source: Aging Cell. 2020 Jun 4;19(7):e13169. doi: 10.1111/acel.13169 (PMC7433010; doi:10.1111/acel.13169)
Supplement: Supplementary file 9 — Supplementary Material [file ACEL-19-e13169-s009.docx]

**Supplementary Information Text**

**Materials and Methods**

***Test compounds and cytotoxicity***

Licochalcone A was purchased from Sigma-Aldrich, St. Louis, MO, USA. In-house LM compounds were synthesized and characterized by NMR spectrum as described (Lee et al., 2012, 2015, 2018). Tet-On ∆K280 Tau_RD_-DsRed 293 and SH-SY5Y cells (Chang et al., 2016) were cultured in Dulbecco’s modified Eagles medium (DMEM) (293) or DMEM-F12 (SH-SY5Y) containing 10% fetal bovine serum (Invitrogen, Carlsbad, CA, USA), 5 μg/ml of blasticidin, and 100 μg/ml of hygromycin (InvivoGen, San Diego, CA, USA). Compound cytotoxicity was assessed by [colorimetric assay](https://en.wikipedia.org/wiki/Colorimetry_(chemical_method)) for measuring cell metabolic activity. Briefly, 5 × 10^4^ cells were plated (48-well plates), grown for 20 h, and treated with the test compounds (0.1-100 µM). After one day, 20 µl of 3-(4,5-dimethylthiazol-2-yl)-2,5-diphenyltetrazolium bromide (MTT, 5 mg/ml in PBS; Sigma-Aldrich) was added to the cells and incubated at 37°C for 3 h. Then 200 μl of lysis buffer (10% Triton X-100, 0.1 N HCl, 18% isopropanol) was added and the absorbance of the insoluble product at OD 570 nm was measured by a microplate reader (FLx800 fluorescence microplate reader, Bio-Tek, Winooski, VT, USA). The half-maximal inhibitory concentration (IC_50_) was calculated using the interpolation method.

***Bioavailability and BBB permeation prediction***

Internet software ChemDraw (<http://www.perkinelmer.com/tw/category/chemdraw>) was used to calculate molecular weight (MW), hydrogen bond donor (HBD), hydrogen bond acceptor (HBA), octanol-water partition coefficient (cLogP), and polar surface area (PSA). In addition, **blood-brain barrier** (BBB) prediction server Online BBB Predictor (<https://www.cbligand.org/BBB/>) (Liu et al., 2014) was used to calculate BBB permeation score.

***His-tagged wild type and*** ***∆K280 pET-28a(+)-Tau_RD_, thioflavin T binding assay, and*** ***transmission electron microscopy (TEM) examination***

Wild type and ∆K280 Tau_RD_ region (+1497~+1883, XM_005275646) with four highly conserved 18-amino acid repeat domains was amplified using the cloned pcDNA5/FRT/TO/Tau_RD_-DsRed (Chang et al., 2016) as templates and synthetic primers 5’-CCATGGATGCAGACAGCCCCCG (*Nco*I site and added ATG codon underlined) and 5’-CTCGAGTTCAATCTTTTTATTTCCTCCG (*Xho*I site underlined). The amplified Tau_RD_-containing fragments were cloned into pGEM-T Easy (Promega Corporation, Fitchburg, WI, USA) and sequenced. The Tau_RD_ fragments were excised with *Nco*I and *Xho*I and subcloned into the corresponding sites of pET-28a(+) (Novagen, Madison, WI, USA). The resulting plasmids were transformed into BL21(DE3)pLysS (Novagen) and His-tagged Tau_RD_ protein expression was induced with 0.1 mM isopropyl-β-D-thiogalactopyranoside (IPTG) for 3 h at 37°C. Bacterial cells were then harvested, and the Tau_RD_-His proteins were purified using His-Bind resins (Novagen) and verified by probing with Tau antibody (1:1000; Santa Cruz Biotechnology).

For thioflavin T binding assay, Tau_RD_ protein (20 μM in final 50 μl) was incubated with tested compounds (1−10 μM) in 150 mM NaCl and 20 mM Tris-HCl, pH 8.0, at 37°C for 48 h to form aggregates. Then thioflavin T (5 μM final concentration; Sigma-Aldrich) was added and incubated for 25 min at room temperature. Thioflavin T fluorescence intensity of samples was recorded by using a microplate reader (Bio-Tek FLx800), with excitation 420 nm and emission 485 nm filter combination.

For Tau_RD_ aggregation examination, the samples of formed fibrils without or with compound addition were placed on a 200-Mesh copper (holey-carbon) grid and viewed on a JEM-1230 transmission electron microscope (JEOL, Tokyo, Japan) at an accelerating voltage of 100 kV.

***∆K280 Tau******_RD_-DsRed fluorescence and thioﬂavin S staining assays***

DsRed fluorescence of proaggregant ∆K280 Tau_RD_-DsRed 293 cells was evaluated to reflect Tau aggregation. On day 1, cells were seeded on a 96-well plate in a density of 0.8 × 10^4^ cells/well. One day after seeding, 0.1-10 µM congo red (as a positive control; Chang et al., 2009) or test compound was added to the cells for 8 h, followed by induction of ∆K280 Tau_RD_-DsRed expression with doxycycline (1 µg/ml; Sigma-Aldrich). On the fifth day, the cells were stained with Hoechst 33342 (0.1 µg/ml; Sigma-Aldrich) for 30 min to identify total cell number; DsRed fluorescence images (543 nm excitation and 593 nm emission) were automatically captured by using the ImageXpressMICRO high-content imaging system (Molecular Devices, Sunnyvale, CA, USA) and analyzed using MetaXpress image acquisition and analysis software (Molecular Devices). In addition, the cells were fixed with paraformaldehyde (4% in PBS) for 15 minutes, permeabilized with methanol (80%) for 6 minutes at -20°C, incubated with thioflavin S (0.02%; Sigma-Aldrich) and 4’,6-diamidino-2-phenylindole (DAPI) (0.1 μg/ml; Sigma-Aldrich) for 5 minutes, and washed in ethanol (50%). Thioflavin S images (FITC 543 nm excitation and 593 nm emission) were captured and analyzed as described. In general, 2 × 10^4^ cells in each biological replicate were analyzed.

***Reactive oxygen species assay***

Cellular reactive oxygen species (ROS) of the above ∆K280 Tau_RD_-DsRed-expressing 293 cells was measured by fluorogenic reagent (CellROX™ Deep Red; Molecular Probes, Eugene, OR, USA) with final concentration of 5 μM and incubated at 37°C for 30 min. The cells were washed with PBS and analyzed by using a flow cytometer (Becton-Dickinson, Franklin Lakes, NJ, USA) with excitation/emission wavelengths of 640/665 nm. For each sample, 5 × 10^4^ cells are analyzed.

***Caspase 3 activity measurement***

∆K280 Tau_RD_-DsRed-expressing cells were lysed in 1 × lysis buffer by repeated cycles of freezing and thawing. Caspase 3 activity was measured with the caspase 3 assay kit according to the manufacturer’s instructions (Sigma-Aldrich).

***Real-time PCR analysis***

Total RNA was extracted using Trizol reagent (Invitrogen). The RNA was reverse-transcribed using SuperScriptTM III reverse transcriptase (Invitrogen). Real-time quantitative PCRs were performed using 50 ng cDNA with a customized Assays-by-Design probe for DsRed (Chang et al., 2017) and TaqMan ﬂuorogenic probes Hs00231713_m1 for CREB, Hs00975961_g1 for NRF2, and 4326321E for HPRT1 (endogenous control) (Applied Biosystems). Fold change was calculated using the formula 2^∆Ct^, ∆C_T_ = C_T_ (control) - C_T_ (target), in which C_T_ indicates cycle threshold.

***Neurite outgrowth analysis***

On day 1, ∆K280 Tau_RD_-DsRed-expressing SH-SY5Y cells were seeded on a 24-well plate in a density of 3 × 10^4^ cells/well, with 10 µM retinoic acid (Sigma-Aldrich) added to induce neuronal differentiation. On the second day, the cells were treated with licochalcone A (1 µM), LM-031 (1 µM), or congo red (10 µM) for 8 h and ∆K280 Tau_RD_-DsRed expression was induced as described. After seven days, the cells were washed with phosphate-buffered saline (PBS) and fixed in 4% paraformaldehyde in PBS for 15 min. After permeabilized with 0.1% Triton X-100 in PBS for 10 min and blocked by 3% BSA in PBS for 20 min, cells were stained with primary neuronal class III β-tubulin (TUBB3) antibody (1:1000; Covance, Princeton, NJ, USA) at 4°C overnight and secondary anti-rabbit Alexa Fluor ®555 antibody (1:1000; Invitrogen) at room temperature for 3 h. After nuclei were stained with DAPI for 30 min, neuronal images were captured using the high-content imaging system as described and analyzed using the Neurite Outgrowth Application Module (Molecular Devices).

***RNA interference***

To knock down CREB expression in ∆K280 Tau_RD_-DsRed SH-SY5Y cells, lentiviral short hairpin RNA (shRNA) targeting CREB (TRCN0000226466), NRF2 (TRCN0000007558), and a negative control scrambled (TRC2.Void) were obtained from National RNAi Core Facility, IMB/GRC, Academia Sinica, Taipei, Taiwan. On day 1, cells were plated on 6-well plates (for protein analysis) or 24-well plates (for neurite outgrowth analysis) in the presence of retinoic acid as described. On the second day, the cells were infected with lentivirus (multiplicity of infection: 3) in medium with polybrene (8 µg/mL; Sigma-Aldrich). On day 3, the culture medium was changed and the cells were pretreated with LM-031 (1 µM) for 8 h, followed by induction of ∆K280 Tau_RD_-DsRed expression. On day 9, the cells were collected for NRF2, CREB, pCREB, and cleaved CASP3 protein analysis or analyzed for neurite outgrowth as described.

***Western blot analysis***

Total proteins from ∆K280 Tau_RD_-DsRed SH-SY5Y cells were obtained using a RIPA buffer containing 50 mM Tris-HCl (pH8.0), 150 mM NaCl, 2 mM EDTA (pH8.0), 50 mM NaF, 0.1% SDS, 0.5% sodium deoxycholate, 1% NP40, and a protease inhibitor cocktail (Sigma-Aldrich). After sonication and sitting on ice for 20 min, the lysates were centrifuged at 14,000 × g for 30 min at 4°C. Protein concentrations were determined using a protein assay kit (Bio-Rad, Hercules, CA, USA), with albumin as a standard. Total proteins (20 µg) were electrophoresed on 10% or 12% SDS-polyacrylamide gel and transferred onto polyvinylidene difluoride (PVDF) membranes (Sigma-Aldrich) through reverse electrophoresis. After being blocked, the membrane was stained with primary antibody against DsRed (1:500; Santa Cruz Biotechnology, Santa Cruz, CA, USA), HSPB1 (1:200; Santa Cruz), NRF2 (1:500; Santa Cruz), NQO1 (1:500; Sigma-Aldrich), GCLC (1:1000; Abcam, Cambridge, MA, USA), CREB (1:500; Santa Cruz), pCREB (S133) (1:1000; Millipore, Billerica, MA, USA), BCL2 (1:500; Santa Cruz), BAX (1:500; BioVision, Milpitas, CA, USA), GADD45B (1:1000; Abcam), BDNF (1:500; Santa Cruz), AKT (1:1000; Abcam), pAKT (S473) (1:500; Cell Signaling, Danvers, MA, USA), ERK1/2 (1:500; Cell Signaling), pERK1/2 (T202/Y204) (1:500; Cell Signaling), CASP3 (1:1000; Cell Signaling), GAPDH (1:1000; MDBio, Taipei, Taiwan), or β-actin (1:5000; Millipore). Subsequently, immune complexes were detected using a horseradish peroxidase-conjugated goat anti-mouse or goat anti-rabbit IgG antibody (1:5000; GeneTex, Irvive, CA, USA) and a chemiluminescent substrate (Millipore).

In addition, isolated hippocampal and cortex tissues from transgenic mice (as described in ***Animal studies***) were homogenized and extracted proteins (10 µg) were separated and blotted. After being blocked, the membranes were probed with antibody against mouse NRF2 (1:500; Sigma-Aldrich), CREB (1:500; Millipore), pCREB (S133) (1:1000; Millipore), or GAPDH (1:1000; MDBio), and the immune complexes detected as described above.

***Parallel artificial membrane permeability assay (PAMPA) to assess BBB permeability of LM-031***

The permeability of LM-031 was determined by a PAMPA-BBB assay. Briefly, the donor well (Millipore) was filled with 300 μl of LM-031 (1 μM) solution. Quality control (QC) compounds, testosterone (high permeability marker), theophylline (low peameability marker), and lucifer yellow (integrity marker) (100 μg/ml) (Sigma-Aldrich), were included for comparison. The filter membrane (PVDF membrane, pore size 0.45 μm; Millipore) was coated with 4 μl of 20 mg/ml porcine polar brain lipid (Avanti Polar Lipids, Alabaster, AL, USA) in dodecane and the acceptor well was filled with 200 μl of acceptor buffer (5% DMSO in PBS). The filter plate was carefully placed on the donor plate to form a “sandwich” (consisting of aqueous donor on the bottom, artificial lipid membrane in the middle, and aqueous acceptor on the top). Each compound was tested in triplicate. The sandwich plate was incubated at room temperature for 18 h. After the permeation time, the PAMPA sandwich plate was separated. The concentration of LM-031 in the acceptor and donor wells was determined using QTRAP 6500 mass spectrometer (AB SCIEX, Singapore) linked to 1200 HPLC system (Agilent Technologies, Palo Alto, CA, USA). The concentration of QC compounds in the acceptor and donor wells was determined using Tecan Infinite M200 Pro micro-plate reader (Switzerland). The effective permeability coefficient (P_e_) was calculated as described ([Ottaviani](https://www.ncbi.nlm.nih.gov/pubmed/?term=Ottaviani%20G%5BAuthor%5D&cauthor=true&cauthor_uid=18620049), Martel, [Escarala](https://www.ncbi.nlm.nih.gov/pubmed/?term=Escarala%20C%5BAuthor%5D&cauthor=true&cauthor_uid=18620049), [Nicolle](https://www.ncbi.nlm.nih.gov/pubmed/?term=Nicolle%20E%5BAuthor%5D&cauthor=true&cauthor_uid=18620049), & [Carrupt](https://www.ncbi.nlm.nih.gov/pubmed/?term=Carrupt%20PA%5BAuthor%5D&cauthor=true&cauthor_uid=18620049), 2008).

***Animal studies***

Mice harboring APP_Swe_, PS1_M146V_, and Tau_P30IL_ transgenes (3×Tg-AD) (Oddo et al., 2003) were purchased from the Jackson Laboratory (004807; Bar Harbor, ME, USA). Six-month-old male homozygous 3×Tg-AD mice were group housed at 20–25°C and 60% relative humidity under a daily 12-h light/12-h dark cycle, with assess to food and water *ad libitum*. Mice were randomly divided into 3 groups: – streptozocin (STZ), STZ, and STZ/LM-031 (*n* = 10 per group). STZ-induced hyperglycemia was used to accelerate the development of AD phenotype (Chen et al., 2014). Briefly, the mice were fasted for 12 h each day prior to intraperitoneal (i.p.) injection of STZ (100 g/kg; Sigma-Aldrich) or vehicle (0.1 M sodium citrate pH4.5) for 4 times (days 2, 3, 9, and 10) (Chen et al., 2019). Mouse body weight was measured and blood glucose level was measured on days 1, 8, 15, 22, and 29 using a blood glucose meter (Bioland Technology, Taipei, Taiwan). LM-031 (40 mg/kg) or vehicle (DMSO:Cremophor EL:0.9% saline = 1:2:7) was intraperitoneally injected every day for 22 days from days 15 to 36. All animal procedures were ethically approved by the Institutional Animal Care and Use Committee of National Taiwan Normal University, Taipei, Taiwan (Permit Number: 103002) and were conducted in compliance with the ARRIVE (Animal Research: Reporting *In Vivo* Experiments) guidelines.

***Open field test***

An open field test was used for monitoring autonomous locomotive activity of the mice. On day 24, each mouse was placed in the center of an open-field apparatus (30 cm long, 30 cm high, and 30 cm wide) and the mouse was allowed to freely explore the box for 10 min. A camera (EDiMAX, Taipei, Taiwan) connected to an automated video tracking system was mounted on the ceiling above the apparatus for collecting data. The total travel (exploratory) distance and rest (inactive) time were analyzed by **PhenoTracker (TSE system,** Thuringia, Germany**).**

***Y-maze task***

A Y-maze composed of three equally spaced arms (40 cm long, 30 cm high, and 15 cm wide) was used to assess spontaneous alternation behavior. On day 26, each mouse was placed in one of the arm compartments and was allowed to move freely for 8 min; the number of entries and the sequence of arms entered were recorded. Spatial working memory was assessed through the spontaneous alternation performances of a Y-maze exploration as described ([Maurice](https://www.ncbi.nlm.nih.gov/pubmed/?term=Maurice%20T%5BAuthor%5D&cauthor=true&cauthor_uid=8069704) et al., 1994). Spontaneous alternation behavior was defined as the entry into all three arms on consecutive choices (correct choice) in overlapping triplet sets. The percent of spontaneous alternation behavior was calculated as the ratio of actual alternation to possible alternations (total arm entries – 2) × 100.

***Morris water maze task***

The water maze apparatus consisted of a circular pool with diameter 100 cm and height 76 cm, a submerged platform 1 cm below the water surface, four types of cues providing distal landmarks in the testing room, and a video camera suspended 250 cm above the center of the pool and connected to a video tracking system. The pool was filled up with water (24–26°C, 35 cm high) rendered opaque by the addition of nontoxic white paint. Mice relied on external cues to find the hidden platform in opaque water. One day prior to spatial training, all mice underwent pretraining to examine animals’ swimming ability and to acclimatize them to the pool. For pretraining (day 29), each mouse was placed in the pool to swim for 60 sec. After three trials, a platform was placed in the center of the pool and the mouse was allowed to remain there for 20 sec. For training (days 30–33), the platform was placed in a quadrant and each mouse received four trials per day for 4 consecutive days, with a cue signaling the platform position. The submerged platform remained there and entry points changed semi-randomly throughout all trials. The trial ended either when the mouse climbed onto the platform or when 60 seconds had elapsed. At the end of each trial the mouse was placed on the platform and faced the cue for 20 sec, learning where the platform was. After 4 training days, all mice were given three testing trials to assess the time taken to climb onto the hidden platform. Then the platform was removed from the pool and each mouse was given two probe trials after 2 and 48 h (days 34 and 36) to record the time spent in the target quadrant for 1 min to assess the retrieval of the short-term and long-term memory about the platform. All the data were analyzed by **PhenoTracker.**

***Immunohistochemistry and image analysis***

Mouse brains were removed, postfixed in 4% paraformaldehyde overnight, and cryopreserved in 30% sucrose solution at 4°C. Series of coronal brain sections (30 µm) were cut in a cryostat (Leica RM2125 RTS, Leica, Wetzlar, Germany). Heat-induced antigen retrieval for immunohistochemistry (IHC) was performed using antigen retrieval buffer (pH9.0, Thermo Fisher Scientific) to break methylene bridges and expose antigenic sites, allowing antibodies to bind. For IHC, brain sections were pretreated with 1% H_2_O_2_ for 15 min to quench endogenous peroxidase activity, and then incubated overnight at 4°C with primary antibody to NeuN, Aβ, or Tau (1:100; Bioss Inc., Woburn, Massachusetts). The sections were then washed and detected by using the UltraVision™ Quanto detection system containing horseradish peroxidase and chromogenic diaminobenzidine substrate (Thermo Fisher Scientific). Sections were counterstained with hematoxylin to stain nuclei (Lab Vision™ Autostainer 480S-2D, Thermo Fisher Scientific), dehydrated in ethanol and xylene, and mounted (Micromount; Leica Biosystems, [Wetzlar](https://en.wikipedia.org/wiki/Wetzlar), Germany) for microscopic examination. IHC toolbox plugin of ImageJ ([Shu](https://www.ncbi.nlm.nih.gov/pubmed/?term=Shu%20J%5BAuthor%5D&cauthor=true&cauthor_uid=24110968) , [Fu](https://www.ncbi.nlm.nih.gov/pubmed/?term=Fu%20H%5BAuthor%5D&cauthor=true&cauthor_uid=24110968) , [Qiu](https://www.ncbi.nlm.nih.gov/pubmed/?term=Qiu%20G%5BAuthor%5D&cauthor=true&cauthor_uid=24110968) , [Kaye](https://www.ncbi.nlm.nih.gov/pubmed/?term=Kaye%20P%5BAuthor%5D&cauthor=true&cauthor_uid=24110968) & [Ilyas,](https://www.ncbi.nlm.nih.gov/pubmed/?term=Ilyas%20M%5BAuthor%5D&cauthor=true&cauthor_uid=24110968) 2013) was used for image processing and analysis. Image was converted into grayscale and adjusted with a threshold to eliminate unwanted areas. After removing non-diaminobenzidine stains, the gray-level intensity and area of the image were collected. To ensure consistency between measurements, the same procedure was applied to all images.

***Statistical analysis***

Data are presented as the mean ± standard deviation of three independent experiments. Differences between groups were evaluated using a two-tailed Student’s *t* test or one-way ANOVA (analysis of variance) with a *post hoc* Tukey test where appropriate. *p* values < 0.05 indicated statistical significance.

**Figure S1. Test compounds.** (a) Solubility (in cell culture medium) of test compounds. (b) Molecular weight (MW), hydrogen bond donor (HBD), hydrogen bond acceptor (HBA), calculated octanol-water partition coefficient (cLogP), polar surface area (PSA), and predicted **blood-brain barrier** (BBB) score of test compounds. (c) Cytotoxicity of test compounds against ∆K280 Tau_RD_-DsRed 293 and SH-SY5Y cells using the MTT assay. Uninduced 293 cells were plated on day 1 and treated with 0.1–10 μM test compounds on day 2. Cell viability was measured on day 5 (n = 3). In addition, uninduced SH-SY5Y cells were seeded with all *trans* retinoic acid (RA; 10 µM) on day 1 and treated with 0.1–10 μM test compounds on day 2. Cell viability was measured on day 9 (n = 3). To normalize, the relative viability in compound untreated cells was set at 100%. IC_50_ values are listed.

**Figure S2.** **ΔK280 Tau_RD_-DsRed 293 cells.** (a) Experimental flow chart. Cells were plated on day 1. On day 2, the cells were treated with congo red or test compounds for 8 h; addition of doxycycline (Dox; 1 µg/ml) followed. DsRed/thioﬂavin S fluorescence, ROS, and caspase 3 activity were assessed on day 5. (b) Cytotoxicity of the test compounds against 293 cells expressing ∆K280 Tau_RD_-DsRed for 3 days using the survived cell number (n = 3). IC_50_ values are listed. (c) Dose response curves of LM-031 based on DsRed fluorescence measurement. (d) Fluorescent images of ΔK280 Tau_RD_-DsRed 293 cells untreated or treated with congo red (10 µM), licochalcone A (1 µM), or LM-031 (1 µM). Nuclei were counterstained with Hoechst 33342 (blue), and merged DsRed and Hoechst 33342 signals were shown. In addition, tau aggregation was monitored by staining with thioﬂavin S (green).

**Figure S3. Tau_RD_-His proteins and Tau** [**aggregation monitored by thioflavin T fluorescence and TEM**](https://r.search.yahoo.com/_ylt=AwrtSXWxN1FdRWwA4SVr1gt.;_ylu=X3oDMTEyMTRzbWdmBGNvbG8DdHcxBHBvcwMyBHZ0aWQDQjg1MzBfMQRzZWMDc3I-/RV=2/RE=1565632561/RO=10/RU=https%3a%2f%2fbio-protocol.org%2fe2941/RK=2/RS=H7upV4aBN5kFZ7ckZYflPdIdwpc-)**.** (a) SDS-PAGE gel (left) and Western blot (right) of wild type (WT) and ΔK280 Tau_RD_ proteins from IPTG-induced bacterial cells. Wild type clone #3 and ΔK280 clone #2 (marked in red) were selected to prepare purified proteins for thioflavin T and TEM studies. (b) Thioflavin T binding assay for Tau_RD_ aggregation. Tau_RD_ protein (20 μM in 50 μl buffer) was incubated with congo red, licochalcone A, or LM compounds (1−10 μM) at 37°C for 48 h, and aggregation was monitored by measuring thioflavin T fluorescence intensity (n = 3). *p* values: comparisons between 0 h and 48 h (^###^: *p* < 0.001), wild type versus ΔK280 (^&&&^: *p* < 0.001), or with and without compound addition (**: *p* < 0.01, ***: *p* < 0.001). (one-way ANOVA with a *post hoc* Tukey test) (c) TEM examination of ΔK280 Tau_RD_ aggregation without or with congo red and LM-004 (10 μM) addition.

**Figure S4. ΔK280 Tau_RD_-DsRed SH-SY5Y cells.** (a) Experimental flow chart. On day 1, the cells were seeded with all *trans* retinoic acid (RA; 10 µM). On day 2, licochalcone A or LM-031 was added to the cells for 8 h, followed by induction of ΔK280 Tau_RD_-DsRed expression with doxycycline (Dox; 1 µg/ml) for 7 days. On day 9, neurite outgrowth and caspase 3 activity were measured. In addition, expression of HSPB1, NRF2, and CREB pathways were assessed. (b) Relative AKT, pAKT, ERK, and pERK protein levels analyzed through immunoblotting. Protein levels were normalized to GAPDH internal control. Relative protein levels are shown on the right side of the representative western blot images. The relative protein level in uninduced cells was normalized (100%). (one-way ANOVA with a *post hoc* Tukey test)

**Figure S5. RNA interference of ΔK280 Tau_RD_-DsRed SH-SY5Y cells.** (a) Experimental flow chart. On day 1, ΔK280 Tau_RD_-DsRed SH-SY5Y cells were plated with retinoic acid (RA; 10 µM). On day 2, the cells were infected with lentivirus-expressing NRF2-specific, CREB-specific, or scrambled shRNA. At 24 h postinfection, LM-031 (1 µM) was added to the cells for 8 h, followed by induction of Tau_RD_-DsRed expression (Dox, 1 µg/ml) for 6 days. On day 9, the cells were collected for NRF2, CREB, pCREB, CASP3, Tau_RD_-DsRed, and neurite outgrowth analyses. (b) NRF2 and CREB mRNA levels relative to endogenous HPRT1 RNA were analyzed by real-time quantitative PCR using gene-speciﬁc ﬂuorogenic probes. To normalize, expression level in uninduced cells was set as 100%. (one-way ANOVA with a *post hoc* Tukey test) (c) Microscopic images of uninduced or induced ΔK280 Tau_RD_-DsRed SH-SY5Y cells with NRF2-specific, CREB-specific, or scrambled shRNA, with or without LM-031 (1 μM) treatments. TUBB3 staining was used to quantify the extent of neurite outgrowth. Nuclei were counterstained with DAPI (blue). Left row, merged TUBB3 (green) and DAPI (blue) signals; right row, outlined images of the neurites and the body (red) for high-content outgrowth quantification.

**Figure S6.** **Permeability of LM-031 and QC compounds testosterone, theophylline, lucifer yellow** **by PAMPA-BBB method.**

**Figure S7.** **Body weight, blood glucose, open field task, NeuN immunoreactivity, and TEM of sarkosyl-insoluble tau in STZ-treated 3×Tg-AD mice.** (a) Body weight and blood glucose of the mice. (b) Open field measurement of spontaneous motor activities (distance traveled) and anxious mood (time inactive) in 10 min of testing period. (c) Representative IHC images for NeuN and intensity quantification in the hippocampus of mice. DG, dentate gyrus; CA1 and CA3, *Cornu Ammonis* areas 1 and 3. (d) TEM of sarkosyl-insoluble tau. *p* values, STZ vs. – STZ mice or STZ/LM-031 vs. STZ mice. *: *p* < 0.05, ^##^: *p* < 0.01, ^###^: *p* < 0.001. (one-way ANOVA with a *post hoc* Tukey test)

**References**

Chang E, Honson NS, Bandyopadhyay B, [Funk KE](https://www.ncbi.nlm.nih.gov/pubmed/?term=Funk%20KE%5BAuthor%5D&cauthor=true&cauthor_uid=19874263), [Jensen JR](https://www.ncbi.nlm.nih.gov/pubmed/?term=Jensen%20JR%5BAuthor%5D&cauthor=true&cauthor_uid=19874263), [Kim S](https://www.ncbi.nlm.nih.gov/pubmed/?term=Kim%20S%5BAuthor%5D&cauthor=true&cauthor_uid=19874263), … [Kuret J](https://www.ncbi.nlm.nih.gov/pubmed/?term=Kuret%20J%5BAuthor%5D&cauthor=true&cauthor_uid=19874263). (2009). Modulation and detec­tion of tau aggregation with small-molecule ligands. *Current Alzheimer Research*, *6*(5), 409–414.

Chang, K. H., Chen, I. C., Lin, H. Y., Chen, H. C., Lin, C. H., Lin, T. H., … Chen, C. M. (2016). [The aqueous extract of *Glycyrrhiza inflata* can upregulate unfolded protein response-mediated chaperones to reduce tau misfolding in cell models of Alzheimer’s disease.](https://www.ncbi.nlm.nih.gov/pubmed/27013866) *Drug Design Development and Therapy*, *10*, 885–896. <https://doi>.org/[10.2147/DDDT.S96454](https://doi.org/10.2147/DDDT.S96454)

Chang, K. H., Lin, C. H., Chen, H. C., Huang, H. Y., Chen, S. L., Lin, T. H., … Yao, C. F. (2017). The potential of indole/indolylquinoline compounds in tau misfolding reduction by enhancement of HSPB1. *CNS Neuroscience & Therapeutics*[,](file:///C:\Users\t4301\Desktop\97-2003夾\,) *23*(1), 45–56. <https://doi.org/10.1111/cns.12592>

Chen, Y., Liang, Z., Tian, Z., Blanchard, J., Dai, C. L., Chalbot, S., … [Gong, C. X](https://www.ncbi.nlm.nih.gov/pubmed/?term=Gong%20CX%5BAuthor%5D&cauthor=true&cauthor_uid=23996345). (2014). Intracerebroventricular streptozotocin exacerbates Alzheimer-like changes of 3×Tg-AD mice. *Molecular Neurobiology*, *49*(1), 547–562. <https://doi.org/10.1007/s12035-013-8539-y>

Chen, Y. C., Chiu, Y. J., Lin, C. H., Hsu, W. C., Wu, J. L., Huang, C. H., …Hsieh-Li, H. M. (2019). [Indole compound NC009-1 augments APOE and TRKA in Alzheimer’s disease cell and mouse models for neuroprotection and cognitive improvement.](https://www.ncbi.nlm.nih.gov/pubmed/30689566) *Journal of Alzheimer’s Disease*, *67*(2), 737–756. https://doi.org/10.3233/JAD-180643

[Lee, Y. T](https://www.ncbi.nlm.nih.gov/pubmed/?term=Lee%20YT%5BAuthor%5D&cauthor=true&cauthor_uid=22772041)., [Jang, Y. J](https://www.ncbi.nlm.nih.gov/pubmed/?term=Jang%20YJ%5BAuthor%5D&cauthor=true&cauthor_uid=22772041)., [Syu, S. E](https://www.ncbi.nlm.nih.gov/pubmed/?term=Syu%20SE%5BAuthor%5D&cauthor=true&cauthor_uid=22772041)., [Chou, S. C](https://www.ncbi.nlm.nih.gov/pubmed/?term=Chou%20SC%5BAuthor%5D&cauthor=true&cauthor_uid=22772041)., [Lee, C. J](https://www.ncbi.nlm.nih.gov/pubmed/?term=Lee%20CJ%5BAuthor%5D&cauthor=true&cauthor_uid=22772041)., & [Lin, W](https://www.ncbi.nlm.nih.gov/pubmed/?term=Lin%20W%5BAuthor%5D&cauthor=true&cauthor_uid=22772041). (2012). Preparation of functional benzofurans and indoles *via* chemoselective intramolecular Wittig reactions. *Chemical communications*[,](file:///C:\Users\t4301\Desktop\97-2003夾\2019-2020資料夾\8%20tau%20WWLm%20嫻%20貞-楨%20美\,) *48*(65), 8135–8137. <https://doi>.org/10.1039/c2cc33972b

[Lee, C. J](https://www.ncbi.nlm.nih.gov/pubmed/?term=Lee%20CJ%5BAuthor%5D&cauthor=true&cauthor_uid=26033344)., [Tsai, C. C](https://www.ncbi.nlm.nih.gov/pubmed/?term=Tsai%20CC%5BAuthor%5D&cauthor=true&cauthor_uid=26033344)., [Hong, S. H](https://www.ncbi.nlm.nih.gov/pubmed/?term=Hong%20SH%5BAuthor%5D&cauthor=true&cauthor_uid=26033344)., [Chang, G. H](https://www.ncbi.nlm.nih.gov/pubmed/?term=Chang%20GH%5BAuthor%5D&cauthor=true&cauthor_uid=26033344)., [Yang, M. C](https://www.ncbi.nlm.nih.gov/pubmed/?term=Yang%20MC%5BAuthor%5D&cauthor=true&cauthor_uid=26033344)., [Möhlmann, L](https://www.ncbi.nlm.nih.gov/pubmed/?term=M%C3%B6hlmann%20L%5BAuthor%5D&cauthor=true&cauthor_uid=26033344)., & Lin, W. (2015). Preparation of furo[3,2-*c*]coumarins from 3-cinnamoyl-4-hydroxy-2H-chromen-2-ones and acyl chlorides: A Bu_3_P-mediated C-acylation/cyclization sequence. *Angewandte Chemie International Edition*[,](file:///C:\Users\t4301\Desktop\97-2003夾\2019-2020資料夾\8%20tau%20WWLm%20嫻%20貞-楨%20美\,) *54*(29), 8502–8505. <https://doi>.org/10.1002/anie.201502789

Lee, S. Y., Chiu, Y. J., Yang, S. M., Chen, C. M., Huang, C. C., Lee-Chen, G. J., … Chang, K. H. (2018). [Novel synthetic chalcone-coumarin hybrid for Aβ aggregation reduction, antioxidation, and neuroprotection.](https://www.ncbi.nlm.nih.gov/pubmed/30596401) *CNS Neuroscience & Therapeutics*, *24*(12), 1286–1298. <https://doi>.org/[10.1111/cns.13058](https://doi.org/10.1111/cns.13058)

[Liu, H](https://www.ncbi.nlm.nih.gov/pubmed/?term=Liu%20H%5BAuthor%5D&cauthor=true&cauthor_uid=24597646)., [Wang, L](https://www.ncbi.nlm.nih.gov/pubmed/?term=Wang%20L%5BAuthor%5D&cauthor=true&cauthor_uid=24597646)., [Lv, M](https://www.ncbi.nlm.nih.gov/pubmed/?term=Lv%20M%5BAuthor%5D&cauthor=true&cauthor_uid=24597646)., [Pei, R](https://www.ncbi.nlm.nih.gov/pubmed/?term=Pei%20R%5BAuthor%5D&cauthor=true&cauthor_uid=24597646)., [Li, P](https://www.ncbi.nlm.nih.gov/pubmed/?term=Li%20P%5BAuthor%5D&cauthor=true&cauthor_uid=24597646)., [Pei, Z](https://www.ncbi.nlm.nih.gov/pubmed/?term=Pei%20Z%5BAuthor%5D&cauthor=true&cauthor_uid=24597646)., ... [Xie, X. Q](https://www.ncbi.nlm.nih.gov/pubmed/?term=Xie%20XQ%5BAuthor%5D&cauthor=true&cauthor_uid=24597646). **(2014).** AlzPlatform: an Alzheimer's disease domain-specific chemogenomics knowledgebase for polypharmacology and target identification research. *Journal of Chemical Information and Modeling*, 2014; 54(4):1050–1060. <https://doi>.org/[10.1021/ci500004h](https://doi.org/10.1021/ci500004h)

[Maurice, T](https://www.ncbi.nlm.nih.gov/pubmed/?term=Maurice%20T%5BAuthor%5D&cauthor=true&cauthor_uid=8069704)., [Hiramatsu, M](https://www.ncbi.nlm.nih.gov/pubmed/?term=Hiramatsu%20M%5BAuthor%5D&cauthor=true&cauthor_uid=8069704)., [Itoh, J](https://www.ncbi.nlm.nih.gov/pubmed/?term=Itoh%20J%5BAuthor%5D&cauthor=true&cauthor_uid=8069704)., [Kameyama, T](https://www.ncbi.nlm.nih.gov/pubmed/?term=Kameyama%20T%5BAuthor%5D&cauthor=true&cauthor_uid=8069704)., [Hasegawa, T](https://www.ncbi.nlm.nih.gov/pubmed/?term=Hasegawa%20T%5BAuthor%5D&cauthor=true&cauthor_uid=8069704)., & [Nabeshima, T](https://www.ncbi.nlm.nih.gov/pubmed/?term=Nabeshima%20T%5BAuthor%5D&cauthor=true&cauthor_uid=8069704). (1994). Behavioral evidence for a modulating role of sigma ligands in memory processes. I. Attenuation of dizocilpine (MK-801)-induced amnesia. [*Brain Research*,](https://www.ncbi.nlm.nih.gov/pubmed/8069704?dopt=Abstract) *647*(1), 44–56. <https://doi>.org/[10.1016/0006-8993(94)91397-8](https://doi.org/10.1016/0006-8993(94)91397-8)

Oddo, S., Caccamo, A., Shepherd, J. D., Murphy, M. P., Golde, T. E., Kayed, R., … [LaFerla, F. M](https://www.ncbi.nlm.nih.gov/pubmed/?term=LaFerla%20FM%5BAuthor%5D&cauthor=true&cauthor_uid=12895417). (2003). Triple-transgenic model of Alzheimer’s disease with plaques and tangles: intracellular Aβ and synaptic dysfunction. *Neuron*, *39*(3), 409–421. <https://doi>.org/[10.1016/s0896-6273(03)00434-3](https://doi.org/10.1016/s0896-6273(03)00434-3)

[Shu, J](https://www.ncbi.nlm.nih.gov/pubmed/?term=Shu%20J%5BAuthor%5D&cauthor=true&cauthor_uid=24110968)., [Fu, H](https://www.ncbi.nlm.nih.gov/pubmed/?term=Fu%20H%5BAuthor%5D&cauthor=true&cauthor_uid=24110968)., [Qiu, G](https://www.ncbi.nlm.nih.gov/pubmed/?term=Qiu%20G%5BAuthor%5D&cauthor=true&cauthor_uid=24110968)., [Kaye, P](https://www.ncbi.nlm.nih.gov/pubmed/?term=Kaye%20P%5BAuthor%5D&cauthor=true&cauthor_uid=24110968)., & [Ilyas, M](https://www.ncbi.nlm.nih.gov/pubmed/?term=Ilyas%20M%5BAuthor%5D&cauthor=true&cauthor_uid=24110968). (2013). Segmenting overlapping cell nuclei in digital histopathology images. ***Conference Proceedings - IEEE Engineering in Medicine and Biology Society***, *2013*, 5445–5448. <https://doi.org/10.1109/EMBC.2013.6610781>
